# Coevolution of species’ range borders: Interactions between interspecific competition, gene flow, and matching habitat choice

**DOI:** 10.1093/evlett/qrag022

**Published:** 2026-06-05

**Authors:** Farshad Shirani, Judith R Miller, Benjamin G Freeman

**Affiliations:** Department of Physics, Emory University, Atlanta, United States; Department of Mathematics and Statistics, Georgetown University, Washington, United States; School of Biological Sciences, Georgia Institute of Technology, Atlanta, United States

**Keywords:** range limits, adaptive dispersal, competitive interactions, non-random gene flow, character displacement

## Abstract

Existing theory examining the coevolutionary dynamics of species’ range borders assumes random dispersal, which causes maladaptive gene flow from the range core to the range margins and contributes to the formation of range limits. However, dispersal is unlikely to be random for many organisms in nature, calling into question existing theoretical predictions. For example, if individuals exhibit phenotype-dependent adaptive dispersal strategies such as matching habitat choice, then the resulting adaptive gene flow toward species’ range margins could facilitate range expansions and potentially prevent the formation of range limits by interspecific competition. To test this idea, we use a comprehensive mathematical model to develop a quantitative theory of range border coevolution that incorporates phenotype-optimal dispersal—a particular form of matching habitat choice in which individuals follow the gradient in an environmental optimum phenotype to settle in the habit best suited for their phenotype. We find that instead of preventing competitively formed range limits, adaptive dispersal leads to sharper range limits and reduced character displacement in sympatry. These differences are particularly remarkable when natural selection is weak, when individuals are specialized in their resource use, or when individuals are highly sensitive to environmental conditions. We show that matching habitat choice causes backward edge-to-core movements, which dynamically interact with the effects of interspecific competition to establish the range limits. Thus, the formation of range limits by interspecific competition is robust to assumptions about individual dispersal. Further, our results identify the competitive advantage of evolving matching habitat choice in steep environmental gradients, especially for slowly growing species in rapidly fluctuating climates.

## Introduction

Understanding the eco-evolutionary processes involved in the evolution of range limits between ecologically similar species is necessary to understand the establishment, structure, and stability of biological communities (Case et al., 2005; Edwards et al., 2018; Urban et al., 2008). The knowledge gained improves our ability to predict how communities will respond to climate change, and enhances management strategies for controlling invasive species (Duputié et al., 2012; Holt & Keitt, 2005; Norberg et al., 2012; Pecl et al., 2017). In principle, every biotic and abiotic factor that affects individuals’ dispersal, adaptation, reproduction, and survival, as well as their interactions with each other and with their environment, will contribute to the evolution of the species’ geographic range and borders (Angert et al., 2020; Mauro et al., 2021; Miller et al., 2020; Sexton et al., 2009; Wisz et al., 2013). A major goal of research has thus been to identify the “key” contributing factors, the presence or absence of which dramatically affects the formation and stability of the range limits. Using a comprehensive mathematical model, we present a theory explaining how interspecific competition interacts with individual dispersal and gene flow to establish borders between two species.

The role of interspecific competition in setting species’ range limits has been investigated for a long time, both empirically (Connell, 1961, 1983; Diamond, 1985; Freeman et al., 2022, 2024; Louthan et al., 2015; Pigot & Tobias, 2013; Schoener, 1983) and theoretically (Case & Taper, 2000; Case et al., 2005; Engen et al., 2021; Goldberg & Lande, 2006; Price & Kirkpatrick, 2009; Shirani & Freeman, 2026; Shirani & Miller, 2022). The work of Case and Taper (2000) stands as a major theoretical contribution. By assuming that species disperse randomly and evolve through natural selection acting on a quantitative trait, they developed a mathematical model that shows how interactions between interspecific competition and maladaptive effects of random gene flow enable the evolution of borders between two species. Although Case and Taper’s results should in general be regarded cautiously—due to a “tenuous” assumption (as they describe it) that they make on species’ trait variance—the main mechanism they describe for coevolution of species’ borders has been reaffirmed by Shirani and Miller (2022) in the absence of any assumption on trait variance.

Case and Taper’s theory relies on the assumption that dispersal is random, and hence creates maladaptive gene flow to range margins (Bonte et al., 2012; Lenormand, 2002, Shirani & Freeman, 2026). However, there is empirical evidence confirming the evolution of adaptive dispersal strategies in a variety of species. The individuals of such species acquire information from their environment to move toward preferred habitat (Clobert et al., 2009; Lowe & McPeek, 2014; Ponchon & Travis, 2022; Ronce, 2007; Saastamoinen et al., 2018). A rather idealized form of such biased movements is *matching habitat choice* (Box 1). The quantitative results by Shirani and Miller (2025) show that matching habitat choice causes the total gene flow to become adaptive, in both central and peripheral populations. Therefore, when species disperse adaptively, Case and Taper’s theory fails to explain whether or not interspecific competition can still result in the evolution of range borders. The uncertainty in the predictions of Case and Taper’s work is amplified by knowing that the analyses performed for a solitary species have revealed that matching habitat choice substantially enhances range expansion capacity and chance of survival when environmental gradients are exceedingly steep (Shirani & Miller, 2025). See Supplementary Section S3 for a summary of key predictions on range evolution of a solitary species. The main goal of the present work is to extend the theory to address these existing uncertainties and describe the eco-evolutionary mechanisms of the formation of range borders under matching habitat choice.

Box 1. Matching habitat choice: concepts and implications

*Matching habitat choice* is a phenotype-dependent adaptive dispersal strategy in which individuals assess their environment and move to the location that best matches their phenotype (Edelaar et al., 2008; Ravigné et al., 2004). In principle, matching habitat choice results in rapid adaptation, reduced within-population (local) genetic variation, and increased between-population genetic divergence. Importantly, matching habitat choice creates directed gene flow that compensates for, or even reverses, the maladaptive effects of random gene flow created by random movements (e.g., to explore the habitat or avoid kin competition) (Edelaar & Bolnick, 2012, 2019; Felsenstein, 1976; Garant et al., 2005; Holt, 1987; Jacob et al., 2017).

**A performance-based strategy:** Matching habitat choice spatially assorts individuals to minimize their phenotype–environment mismatch. Thus, matching habitat choice is in fact a utility- or performance-based strategy (Edelaar et al., 2008; Munar-Delgado et al., 2024; Ravigné et al., 2004). Although maximizing individuals’ performance likely increases their fitness and overall population fitness as well, it is important to note that matching habitat choice is not in principle a fitness-maximizing (ideal free) dispersal strategy. Many factors that affect population fitness, such as competition, do not directly contribute to individuals’ search for a phenotype matching environment (Shirani & Miller, 2025).

**Dependence on niche breadth:** An individual’s performance in a local environment depends on how close the conditions of the environment are to the conditions at the core of the individual’s niche (Holt, 2009). Further, it is reasonable to imagine that adaptively dispersing individuals have a fairly accurate perception of their own niche breadth. Thus, the operation of matching habitat choice depends strongly on individuals’ niche breadth, that is, the broadness of the range of resources the individuals can utilize or the environmental conditions they can tolerate. The narrower the individuals’ niche breadth, the stronger their potential to disperse to a matching habitat.

**(In)dependence on natural selection:** Even though natural selection is involved in the evolution of matching habitat choice, the operation of matching habitat choice is not driven by natural selection (Edelaar et al., 2023). In fact, it is unlikely that an individual would be able to develop a perception of the strength of natural selection in order to decide whether or not it should move to another place.

**Conflicting effects with competition:** Matching habitat choice and competition have conflicting effects on adaptation and phenotypic variation. Phenotype-dependent competition between individuals creates a frequency-dependent diversifying (disruptive) selection, which in general tends to increase local trait variance. When individuals specialize in utilizing resources based on their phenotype, the farther the phenotypes are from each other the less intense the average level of competition is within the population, hence the less loss of population fitness (growth rate). By contrast, matching habitat choice tends to spatially assort the phenotypes, bringing similar phenotypes close together. This substantially reduces local trait variation and intensifies competition between phenotypes, which can then compromise the adaptive effects of matching habitat choice on population fitness.

Phenotype-dependent competition and matching habitat choice have conflicting effects on adaptation and phenotypic variation (Box 1), which makes predicting their joint contribution to range evolution a challenging problem. Including further the effects of natural selection, density-dependent effects of gene flow, and interactions with environmental gradients, makes the resulting coevolutionary range dynamics hard to predict intuitively. The mathematical framework of the model we use in the present work allows for quantifying each such effect, thereby providing a mechanistic explanation of how they interact to form range borders. The model, built on the models developed by Shirani and Miller (2022, 2025) (which are based on the seminal works of Case & Taper, 2000, and Kirkpatrick & Barton, 1997), includes all the ingredients necessary to establish a quantitative theory: an environmental gradient in trait optimum, density-dependent population growth, Allee effect, directional and stabilizing selection, mutational changes in frequency of the phenotypes, phenotype-dependent competition, random dispersal, and *phenotype-optimal dispersal*—a special and possibly more realistic type of matching habitat choice (Box 2).

We develop quantitative results that explain how range borders evolve through the interactions between interspecific competition, matching habitat choice, gene flow, environmental gradients, and natural selection. We show that directed movements and the adaptive gene flow they create make the mechanism of border formation essentially different from the case where dispersal is only random. We explore the effects of key parameters such as strength of matching habitat choice, steepness of the environmental gradient, strength of natural selection, and degree of individuals’ specialization on the range borders formed between the species. Specifically, we show how these parameters affect the sharpness of the range borders and the extent of character displacement that the species exhibit where they overlap. We finally demonstrate how an adaptive dispersal strategy such as matching habitat choice confers a competitive advantage upon a species, particularly in rapidly and frequently fluctuating environments.

## Methods

Our theoretical discussions are based on the results we obtain by numerically solving the equations of our mathematical model. The model is composed of a system of partial differential equations, representing the joint evolution of the population density and the mean and variance of a quantitative phenotypic trait for each of the species present in a community of competitively interacting species. The mathematical description of the model is provided entirely in Supplementary Section S1. Below, we only provide a brief overview of the structure of the model. Throughout the present work, we only focus on coevolution of range borders between two species, even though the equations of the model in Supplementary Section S1 are presented for the general case of a community with an arbitrary number of species. In presenting the mathematical formulations, we follow the notations used by Shirani and Miller (2022, 2025). To prevent potential misunderstandings, in Supplementary Table S2 we provide a list of notational differences compared to previous models in the literature.

Individuals of each species *i*, $i=1,2$, in the model are represented by a fitness-related quantitative phenotypic trait, which is a random variable taking any value in $\mathbb {R}$. The model has a quantitative phenotypic framework. The populations are assumed to be fully polymorphic, meaning that all phenotype values are assumed to be present in the populations at all times. The main equations of the model (Supplementary Equations (S1)– (S3)) along with their nonlinear terms defined by Supplementary Equations (S4)–(S14) represent the joint evolution of species’ population density, $n_i(x,t)$, $i=1,2$, their trait mean $q_i(x,t)$, and their trait variance $v_i(x,t)$, where *x* denotes a point in the geographic space and *t* denotes an instance of time. For populations with normally distributed phenotypes, as we assume in our model (Assumption (iv) in Supplementary Section S1.4), the variables $n_i$, $q_i$, and $v_i$ essentially capture all that is needed to represent the populations dynamics. The parameters of the model and their range of values are given in Supplementary Table S1. The choices of units for the quantities involved in the model are described in Supplementary Section S1.3. The units of time, space, trait values, and population abundances are denoted by $\mathtt {T}$, $\mathtt {X}$, $\mathtt {Q}$, and $\mathtt {N}$, respectively. The default value $\mathrm{R}_i = 1 \,\, \mathtt {T}^{-1}$ chosen for the maximum intrinsic growth rate of the populations in our simulations represents slowly growing species such as birds (Niel & Lebreton, 2005), for which the evolution of matching habitat choice is likely more beneficial (Shirani & Miller, 2025).

Box 2. Matching habitat choice: a continuum model

Following Shirani and Miller (2025), we use the term *phenotype-optimal dispersal* to refer to a special type of matching habitat choice in which individuals follow the gradient in trait optimum to eventually settle in a habitat location that minimizes their phenotype–environment mismatch. We use the continuum model of phenotype-optimal dispersal proposed by Shirani and Miller to incorporate matching habitat choice into the model of competitively interacting species we use in our study.

**Phenotypic potential energy:** To model the force perceived by individuals to disperse to matching habitats, Shirani and Miller define a *phenotypic potential energy* function, a quantitative measure of individuals’ self-assessment of (mis)performance. Denoted by $\theta _i(x,p)$, the phenotypic potential energy of phenotype *p* in the *i*th species at geographic location *x* is defined by the following simplified yet meaningful function,

1 \begin{eqnarray*}
\theta _i(x,p) := \frac{\big (p - \mathrm{Q}(x)\big )^2}{2 \mathrm{V}_i}, \quad \quad i=1, 2.
\end{eqnarray*}

This scalar potential energy function provides a quantitative measure of how poorly the phenotype *p* is adapted to (can perform at) location *x*. Or, equivalently, $\theta _i(x,p)$ determines how poorly the phenotype *p* can utilize the resources that are most favorable for phenotypes with the optimum value $\mathrm{Q}(x)$. Consistent with the concepts of matching habitat choice described in Box 1, this phenotypic potential is independent of the strength of natural selection. It depends on the degree of individuals’ phenotype–environment mismatch and the within-phenotype component of the species’ niche breadth. The latter is quantified by the variance of the individuals’ phenotype/resource utilization distributions $\mathrm{V}_i$ (Ackerly & Cornwell, 2007; Shirani & Miller, 2022; Violle & Jiang, 2009). See Supplementary Section S1.5 for further details.

**Phenotypic dispersal force:** The phenotypic potential energy perceived by the individuals induces a *phenotypic dispersal force*,

2 \begin{eqnarray*}
-\nabla _x\theta _i(x,p) = \frac{p - \mathrm{Q}(x)}{\mathrm{V}_i} \, \nabla _x\mathrm{Q}(x), \quad i=1, 2,
\end{eqnarray*}

which directs the movements of phenotype *p* along the environmental gradient $\nabla _x\mathrm{Q}(x)$ toward a better-matching habitat location.

**Perceived environmental gradient:** A biological organism is unlikely to develop a perception of the environmental gradient in an exact mathematical sense. Further, the magnitude of the dispersal force perceived by an individual is unlikely to remain directly proportional to the steepness of the environmental gradient (as in Equation (2)) when the gradient becomes increasingly steep. Therefore, when incorporating Equation (2) in the model of phenotype-optimal dispersal, the environmental gradient $\nabla _x\mathrm{Q}$ is replaced with its perceived value $\widetilde{\nabla _x\mathrm{Q}}$. A simplified model is used for the perceived gradient such that its direction is always the same as the direction of the actual gradient but its magnitude (steepness) saturates to a maximum value when the actual gradient becomes increasingly steep. See Supplementary Section S1.5 and Shirani and Miller (2025, Remark 3) for further details.

**A niche-dependent model of phenotype-optimal dispersal:** Using the dispersal force (Equation (2)), the phenotype-optimal dispersal of a phenotype *p* in the *i*th species is modeled by advective (directed) movements, the velocity of which is given by $\mathrm{A}_i(x) (-\nabla _x\theta _i(x,p))$, $i=1,2$. The parameter $\mathrm{A}_i$, which we assume to be constant in space (and time) throughout this work, denotes the perceived *propensity* of the individual to disperse optimally. Setting $\mathrm{A}_i = 0$ in our analyses makes dispersal entirely random. Non-zero values of $\mathrm{A}_i$ include optimal dispersal, in addition to random dispersal which is always present in the model. The greater the value of $\mathrm{A}_i$, the stronger the effects of optimal dispersal.

At each location *x*, the density of phenotypes changes as a result of four different processes: random dispersal, phenotype-optimal dispersal (Box 2), intrinsic growth of the population, and mutation; see Supplementary Section S1.5.The mathematical representation of these processes requires making certain assumptions, a list of which is provided in Supplementary Section S1.4. The intrinsic growth rate of the phenotypes depends on the intensity of competition between them, the strength of natural selection, and Allee effect. The intensity of competition between individuals depends on how close their phenotype values are to each other, and is further adjusted by individuals’ *phenotype utilization variance*$\mathrm{V}_i$, $i=1,2$; see Supplementary Section S1.5. The default value $\mathrm{V}_i = 4\,\, \mathtt {Q}^2$ in Supplementary Table S1 results in fairly strong interspecific competition, noting that the per capita effects of competition between phenotypes follow a Gaussian form with variance $2 \mathrm{V}_i$ when $\mathrm{V}_i$ takes the same value for both species; see the competition kernel given by Supplementary Equation S20. Natural selection penalizes the phenotype values that are different from the environmental optimum phenotype $\mathrm{Q}(x)$. We assume $\mathrm{Q}$ changes linearly in space, representing an environmental gradient $\nabla _x\mathrm{Q}$ with constant steepness.

The model includes both random and phenotype-optimal dispersal. The random component can represent the exploratory movements that individuals may initially perform to assess the environment and move to a matching location. This component can also represent any other uninformed movements to escape kin competition or avoid inbreeding. The phenotype-optimal component is modeled as described in Box 2. The strength of this dispersal component depends on individuals’ perceived *propensity* to disperse adaptively, denoted by parameter $\mathrm{A}_i$ for the *i*th species. Setting $\mathrm{A}_i = 0$ makes dispersal entirely random. Non-zero values of $\mathrm{A}_i$ then include optimal dispersal, in addition to the always present random dispersal.

## Results

To obtain our numerical results, we consider a one-dimensional habitat with reflecting boundaries, wherein the two populations are initially distributed allopatrically. The details of population initialization are described in Supplementary Section S2.2. We assume the trait optimum $\mathrm{Q}$ increases linearly along the habitat. Using this simulation layout, we numerically solve Supplementary Equations (S1)–(S14) with parameter values specified independently for each simulation.

**Figure 1.**
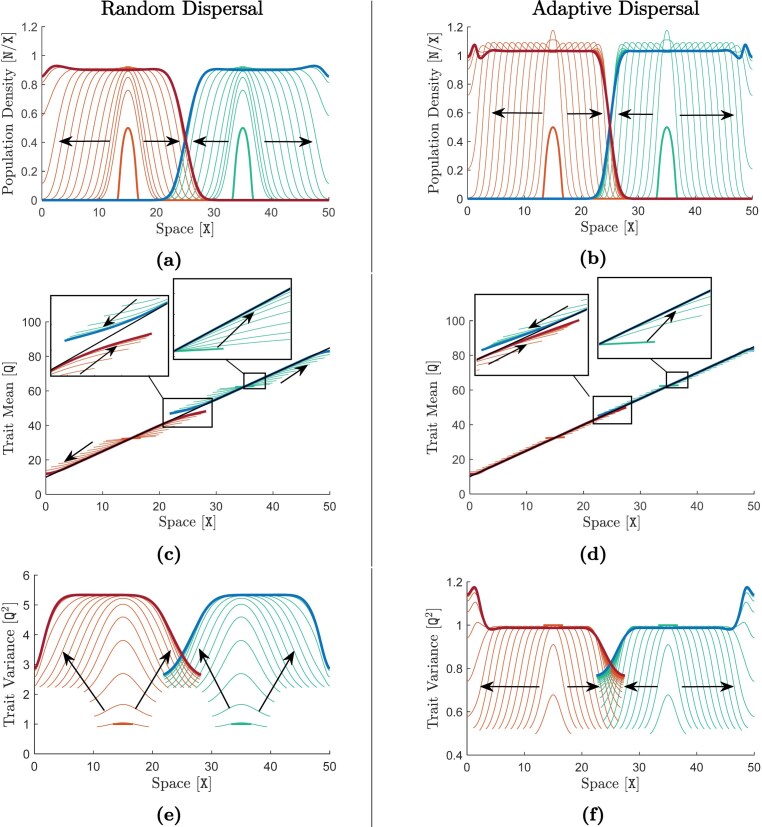
Coevolution of species’ range borders and formation of a region of sympatric coexistence. The two species simulated in the left panel (graphs (a), (c), and (e)) disperse only randomly, that is, $\mathrm{A}_1 = \mathrm{A}_2 = 0 \,\, \mathtt {X}^2/\mathtt {T}$. In the right panel, the two species perform strong phenotype-optimal dispersal with $\mathrm{A}_1 = \mathrm{A}_2 = 10 \,\, \mathtt {X}^2/\mathtt {T}$. The rest of the parameters of the two species in both panels are set equal to the default values given in Supplementary Table S1. The trait optimum $\mathrm{Q}$ is linear and increasing, shown by the black line in panels (c) and (d), with a steep gradient of $\nabla _x\mathrm{Q}= 1.5 \,\, \mathtt {Q}/\mathtt {X}$. Curves of the species’ population density $n_i$, as their range evolves in time, are shown in panels (a) and (b). Curves of the species’ trait mean $q_i$ are shown in panels (c) and (d). Curves of the species’ trait variance $v_i$ are shown in panels (e) and (f). Note the difference in the scales of the y-axis in panels (e) and (f). In all graphs, curves are shown at every $4\,\, \mathtt {T}$ for a simulation time horizon of $T = 300 \, \mathtt {T}$, at which an equilibrium state is approximately formed. For the first (left) species, curves are shown in orange, thick orange curves indicate the initial curves at $t=0 \, \mathtt {T}$, and the final curves at the end of the simulation are highlighted in red. For the second (right) species, curves are shown in green, thick green curves indicate the initial curves, and the final curves are highlighted in blue. Arrows show the direction of evolution in time. Although the solutions of the model are computed for the entire habitat, the values of trait mean and trait variance outside the range of the species are not biologically meaningful. Therefore, the portions of the curves that are outside the species’ effective ranges (i.e., the regions where $n_i < 0.02$, $i=1,2$) are not shown in panels (c)–(f). Graphs (a) and (b) show that the species’ ranges converge to an equilibrium state, at which a limited region of sympatry is formed at the interface of the two species in the middle of the habitat. The insets in panels (c) and (d) highlight the formation of character displacement over the regions of sympatry, as well as the speed of species’ adaptation (convergence to the trait optimum) to the environment.

### Coevolution of species’ range borders

Figure 1 shows evolutionary population dynamics of the species in a steep environmental gradient, both in the absence (left panel) and in the presence (right panel) of phenotype-optimal dispersal. When dispersal is only random, the coevolution of the borders follows the existing theory (Case & Taper, 2000; Shirani & Miller, 2022). The initial populations gradually adapt ($q_i \rightarrow \mathrm{Q}$) to new locations and expand their range. The curves of trait mean in Figure 1c show significant maladaptation near range margins, where the curves are flattened and fail to follow the slope of the gradient. This is due to the maladaptive effects of asymmetric core-to-edge gene flow. As a result, trait variance also declines at range margins (Figure 1e); see the discussions by Shirani and Miller (2022) for further details. At the time the two populations meet at the center of the habitat and become sympatric over a short region, they are both fairly well-adapted to the environment. Therefore, a strong competition is initiated between them, which induces character displacement (hence departure of $q_1$ and $q_2$ from $\mathrm{Q}$) in their overlapping subpopulations. The resulting maladaptation along with the direct fitness loss caused by competition substantially reduces the density of the populations at their interface. As a result, the asymmetry in core-to-edge gene flow within each species is intensified, moving the species’ trait mean over the region of sympatry further away from the optimum (increasing character displacement). As range edges advance further and the spatial overlap between the species grows, this reinforcing feedback between competitively induced character displacement and maladaptive gene flow continues to create stronger levels of maladaptation at range edges. It eventually prevents local adaptation at edges and halts range expansions, resulting in the formation of range borders (Figure 1a).

**Figure 2.**
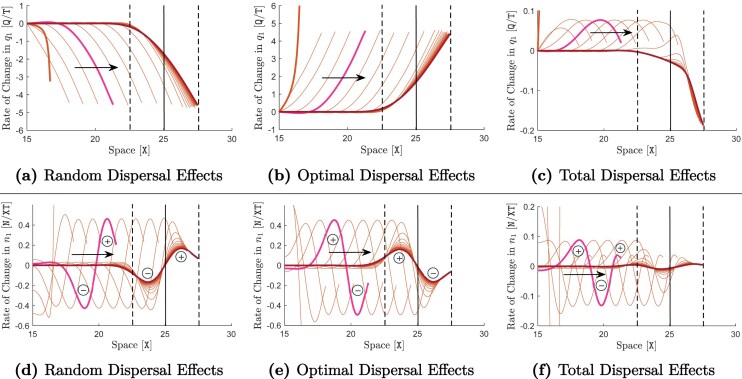
Effects of random and optimal dispersal on rates of change in trait mean and population density. The graphs shown here are associated with the same simulation performed for the results shown in the right panel of Figure 1, that is, with $\mathrm{A}_1 = \mathrm{A}_2 = 10 \,\, \mathtt {X}^2/\mathtt {T}$ and $\nabla _x\mathrm{Q}= 1.5 \,\, \mathtt {Q}/\mathtt {X}$. Similar graphs but associated with the left panel of Figure 1 ($\mathrm{A}_1 = \mathrm{A}_2 = 0 \,\, \mathtt {X}^2/\mathtt {T}$) are shown in Supplementary Figure S2. The contributions of random dispersal (random gene flow), phenotype-optimal dispersal (directed gene flow), and total dispersal (net gene flow) to the rate of change of trait mean (adaptation or maladaptation rate) within the first species, $\partial _tq_1$, are shown in graphs (a)–(c) in the upper panel. The contributions of these dispersal components to the rate of change of population density of the first species, $\partial _tn_1$, are shown in the lower panel. Note the difference in the scales of the y-axis in all graphs. The details of the computations associated with these contributions are described in Supplementary Section S2.3. The curves in all graphs are shown only for the right half of the species’ range, where it meets and coevolves with the second species. The curves for the second species are shown in Supplementary Figure S1. In all graphs, curves are shown at every $4 \,\, \mathtt {T}$, and the portions of the curves that lie outside the species’ range are not shown. The final equilibrium curves obtained (approximately) at the end of the simulation ($T=300 \,\, \mathtt {T}$) are highlighted in red. The sample curves highlighted in pink are associated with the species’ range expansion regime before meeting the second species. The solid black lines indicate the center of the habitat where the interface between the two species (Figure 1b) is formed. The dashed lines indicate the boundaries of the region of sympatry formed at the interface.

**Figure 3.**
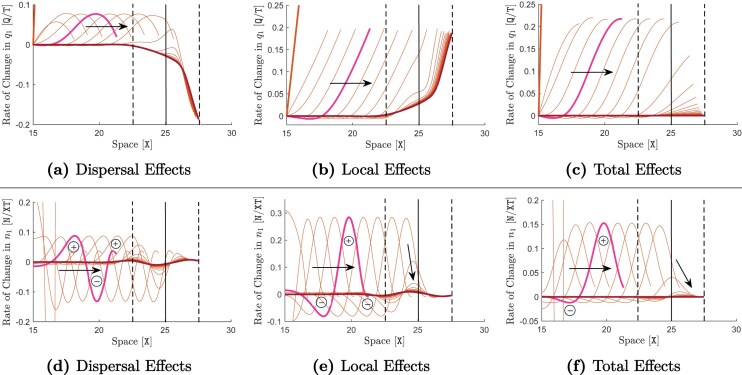
Effects of adaptive dispersal, selection, and competition on rates of change in trait mean and population density. The graphs shown here complete the set of graphs shown in Figure 2 to demonstrate the effects of different components contributing to $\partial _tq_1$ (top panel) and $\partial _tn_1$ (bottom panel). As in Figure 2, the graphs are associated with the same simulation shown in the right panel of Figure 1, that is, with adaptive dispersal. Similar graphs but associated with the left panel of Figure 1 (random-only dispersal) are shown in Supplementary Figure S3. Graphs (a) and (d) are the same graphs shown in Figure 2c and f, which are repeated here for simplicity of comparison. They show the contribution of total adaptive dispersal (random plus phenotype-optimal) to $\partial _tq_1$ and $\partial _tn_1$, respectively. The local contributions of selection and competition to $\partial _tq_1$ and $\partial _tn_1$ are shown in panels (b) and (e), respectively. The details of the computations associated with these contributions are described in Supplementary Section S2.3. Graphs (c) and (d) show the total effects. That is, panel (c) shows exactly the curves of $\partial _tq_1$, obtained as the sum of the curves in panels (a) and (b). Similarly, panel (f) shows exactly the curves of $\partial _tn_1$, obtained as the sum of the curves in panels (d) and (e). The same description as in Figure 2 holds for the curve colors and the solid and dashed lines. Note the difference in the scales of the y-axis in all graphs.

The results shown in Figure 1 confirm that range borders are also evolved in the presence of adaptive dispersal. However, compared with the random dispersal case, we observe several distinctive differences. Specifically, the region of sympatry at equilibrium is shorter and species’ borders are sharper (Figure 1b). The species’ population density presents a small overshoot near range edges, which disappears at equilibrium. Adaptation occurs much more rapidly, and the extent of character displacement at equilibrium is substantially smaller (insets in Figure 1d). Finally, the local trait variance is dramatically lower (Figure 1f). To understand the causes of these differences and the underlying mechanism of the formation of range borders, we compute the contribution of each component of dispersal to the rates of changes in species’ trait mean, $\partial _tq_i$, and species’ population density, $\partial _tn_i$, $i=1,2$. We also compute the local contributions to $\partial _tq_i$ and $\partial _tn_i$ due to the effects of selection and competition. The details of these computations are provided in Supplementary Section S2.3. The results are shown in Figures 2 and 3 for the first species and in Supplementary Figure S1 for the second species.

The contributions of the dispersal components to $\partial _tq_1$ confirm strong adaptive effects of the directed gene flow created by phenotype-optimal dispersal (Figure 2a–c). To see these effects, first note that positive values of $\partial _tq_1$ at a location *x* imply that the species’ trait mean increases at *x*. Contrariwise, negative values of $\partial _tq_1$ imply a decrease in the trait mean. Also, note that the curves in Figure 2 are shown for the right half of the first species range, in which the initial profile of the species’ trait mean lies below the trait optimum; see Figure 1d. Therefore, positive values of $\partial _tq_1$ imply adaptive effects (increasing $q_1$ toward $\mathrm{Q}$) and negative values imply maladaptive effects. Based on these observations, Figure 2 confirms that the random gene flow created by random dispersal is always maladaptive, particularly at range margins (wavefronts)—a result consistent with the known disruptive effects of random gene flow (Kirkpatrick & Barton, 1997; Lenormand, 2002; Shirani & Freeman, 2025). By contrast, Figure 2b shows that the directed gene flow created by matching habitat choice is always adaptive, even at range margins. Further, it almost completely compensates for the maladaptive effects of random gene flow. As a result, the total gene flow caused by overall dispersal becomes adaptive, except over a short region near the range edge at equilibrium (see the red curve in Figure 2c). The creation of this adaptive gene flow explains the rapid adaptation shown in Figure 1d. It also implies that disruptive gene flow does not play a major role in forming borders when species disperse adaptively. We note that the maladaptive effects observed in Figure 2c near the range edge at equilibrium are much weaker than the swamping effects observed in the absence of matching habitat choice; see Supplementary Figure S2c. We note further that natural selection also acts locally to adapt the populations (Figure 3b). However, the adaptation caused by selection in the presence of phenotype-optimal dispersal is much slower than the adaptation caused when dispersal is only random (Supplementary Figure S3b). This is because the phenotype–environment matching caused by phenotype-optimal dispersal substantially removes the need for selection to act.

When dispersal is only random, movements near the range margins are strongly asymmetric, predominantly from the populous center of the species to their sparsely populated periphery—hence creating the asymmetric core-to-edge gene flow we discussed before. These asymmetric movements are schematically illustrated in Supplementary Figure S4a. They can be seen more accurately through the spatial profile of the contribution of dispersal to $\partial _tn_i$, shown for the first species in Supplementary Figure S2f. This profile is also approximately preserved in the contribution of the random component of dispersal in the presence of optimal dispersal; see Figure 2d and Supplementary Figure S1d. At the wavefront of the species’ population density, where the density declines from its maximum (at core) to almost zero (at edge), the contribution of random dispersal to $\partial _tn_1$ shows a transition from negative values (near the core) to positive values (near the edge). This $(-)(+)$ transition pattern can be seen in Supplementary Figure S2f and is present both during the range expansion regime and at equilibrium. The transition point from $(-)$ to $(+)$ occurs at the inflection point on the wavefront of the curve of population density in Figure 1a. The negative values of $\partial _tn_1$ in the core side of the wavefront implies that the “overall” random movement over that region is outward, that is, toward the edge. As a result, the contribution of random dispersal to $\partial _tn_1$ becomes positive (increasing the density) near the edge. This confirms that interspecific competition and the strong migration load caused by random gene flow are then the factors which act locally (Supplementary Figure S3e) to canceling out the density-increasing effects of random movements near the edge, permitting the establishment of borders at equilibrium. This cancellation can be seen through Supplementary Figure S3d–f.

Figure 2d–f demonstrates a key process contributing to the coevolution of range borders in the presence of matching habitat choice: backward edge-to-core movements that completely compensate for, and even partially reverse the effects of core-to-edge movements caused by random dispersal; see also the illustration in Supplementary Figure S4. The overall edge-to-core movement is implied by the $(+)(-)$ pattern in the contribution of adaptive dispersal to $\partial _tn_1$ at wavefronts (Figure 2e), which is opposite to the $(-)(+)$ pattern of contribution of random dispersal. When the individuals at the core randomly move to or beyond the range edge, for example to explore their surroundings during range expansion, the majority of them find the new habitat less suitable for their phenotype. Matching habitat choice then pushes them to move back to the core. These backward movements at wavefronts underlie several of the distinctive differences we observed in Figure 1 in comparison with the case of random-only dispersal. During the range expansion regime, the backward movements cause the contribution of overall dispersal to $\partial _tn_1$ to follow a $(+)(-)(+)$ pattern at wavefronts (Figure 2f). Similar to the case of random-only dispersal (Supplementary Figure S3d and e), the local contribution of competition and selection to $\partial _tn_1$ then follows an opposite pattern $(-)(+)(-)$; see Figure 3d and e. This results in the complete profile of $\partial _tn_1$ to become slightly negative near the core before transitioning to positive at the wavefronts (Figure 3f), explaining the overshoots observed in range expansion waves in Figure 1b. It is worth noting that the patterns we see in the contributing components to $\partial _tn_1$ remain qualitatively unchanged when gene flow is predominantly directed (optimal). We made this observation by simulating an artificially strong level of phenotype-optimal dispersal with $\mathrm{A}_i = 50 \,\, \mathtt {X}^2/\mathtt {T}$, that is not shown here.

When the two species meet, interspecific competition and backward edge-to-core movements induced by matching habitat choice interact to establish the range limits (Supplementary Figure S4b). Interspecific competition tends to induce character displacement over the region of sympatry gradually formed between the species. However, character displacement creates a phenotype–environment mismatch in the sympatric subpopulations. Matching habitat choice then pushes the individuals at range margins to move back to better-matching locations at the core, hence reducing the extent of character displacement. These interactions between interspecific competition and backward movements eventually reach a balanced steady state, with significantly reduced character displacement compared with the case of random-only dispersal. The evolving curves in Figures 1b, 1d, 2, and 3 confirm the presence and convergence to this equilibrium state. At equilibrium, the overall edge-to-core movement resulting from adaptive dispersal almost completely cancels out the core-to-edge random movements, leading to almost no density change by total dispersal (Figure 2f)—a dramatic difference compared with the random-only dispersal case (Supplementary Figure S2f). This also explains the enhanced sharpness of the borders in the presence of adaptive dispersal (Figure 1b). Since overall dispersal does not significantly contribute to density change at equilibrium, the density decline from core to edge near the borders is predominantly due to intensified interspecific competition that acts locally in space, as well as the slight maladaptive gene flow that is created only close to range edges at equilibrium (Figure 2c).

### Effects of dispersal, environment, and specialization on character displacement and sharpness of the borders

**Figure 4.**
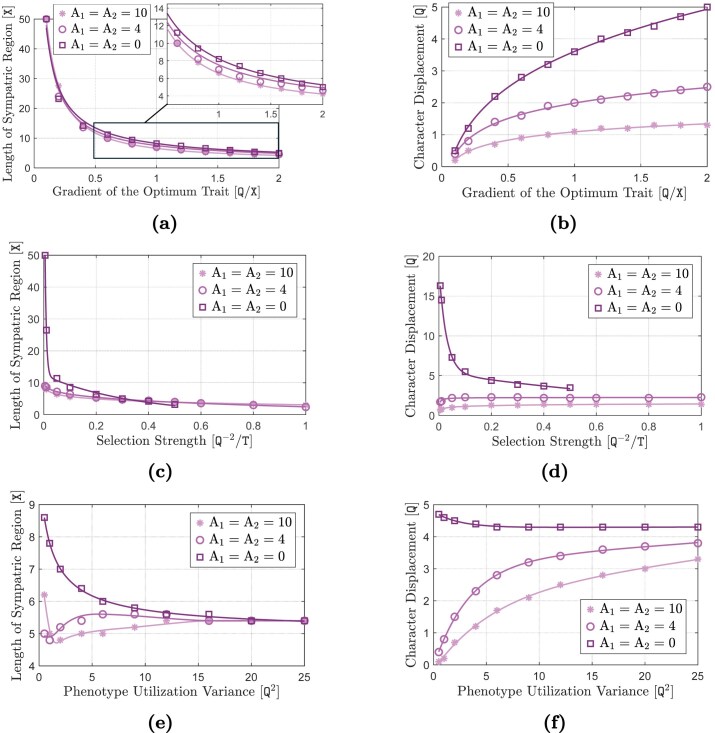
Effects of matching habitat choice, gradient steepness, selection strength, and individuals’ specialization on character displacement and region of sympatry at equilibrium. The lengths of the region of sympatry when species’ ranges converge to an equilibrium state (e.g., as in Figure 1c and d) are shown in panels (a), (c), and (e). Correspondingly, the extents of character displacement at equilibrium are shown in panels (b), (d), and (f). In the upper, middle, and lower panels, the results are shown, respectively, for a broad range of values of the steepness of the environmental gradient, the strength of the stabilizing selection ($\mathrm{S}$), and the variance of the phenotype (resource) utilization distribution (assuming $\mathrm{V}_1 = \mathrm{V}_2$). In all graphs, the results are shown for three different values of the optimal dispersal propensity, with $\mathrm{A}_1 = \mathrm{A}_2= 0 \,\, \mathtt {X}^2/\mathtt {T}$ resulting in only random dispersal. The environmental gradient for the results shown in the middle and lower panels is set to be steep, $\nabla _x\mathrm{Q}= 1.5 \,\, \mathtt {Q}/\mathtt {X}$. The rest of the parameters take their default values given in Supplementary Table S1. The data points are obtained as follows. At different values of the variable parameter, a simulation similar to those associated with Figure 1 is performed for a period of time long enough for species’ range evolution to converge to a steady state (equilibrium). In each simulation, the region of sympatry is identified as the region over which both species coexist with a density greater than $0.02 \,\, \mathtt {N}/\mathtt {X}$. The lengths of the identified regions are marked by the data points in panels (a), (c), and (e). Denoting the equilibrium trait mean of the *i*th species by $q_i^{\ast }$, the extent of character displacement is computed as the maximum value of $|q_1^{\ast }(x) - q_2^{\ast }(x)|$ when *x* takes all values in the region of sympatry. In our simulations, the maximum character displacement always occurs at the species’ range boundary. The computed character displacements are marked by the data points in panels (b), (d), and (f). The curves are obtained by interpolating the data points. We note that, in the absence of optimal dispersal ($\mathrm{A}_1 = \mathrm{A}_2 = 0$), the species’ range expansion becomes too slow when $\mathrm{S}$ takes values greater than $0.5 \,\, \mathtt {Q}^{-2}/\mathtt {T}$. In this case, the species eventually become extinct when $\mathrm{S}\gtrsim 0.88 \,\, \mathtt {Q}^{-2}/\mathtt {T}$, the critical value that can be computed using the formula given by Shirani and Miller (2022, Remark 4).

We explore further the differences between the coevolution of borders under matching habitat choice and under random dispersal, by computing the extent of character displacement and the length of the region of sympatry at equilibrium. We perform computations for random-only and moderate and strong optimal dispersal propensity, as well as broad ranges of values for the steepness of the environmental gradient, the strength of stabilization selection, and the individuals’ specialization level. The results are shown in Figure 4.

Figure 4a shows that the region of sympatry expands when the environmental gradient becomes shallower. When the gradient is sufficiently shallow ($\nabla _x\mathrm{Q}\rightarrow 0$), the species become sympatric over the entire habit and no borders are formed between them. This is because in shallow gradients the maladaptive core-to-edge effects of random dispersal do not become strong enough to destabilize the competition-selection balance at range margins and set range limits. The state of full sympatry evolves as $\nabla _x\mathrm{Q}\rightarrow 0$ regardless of the strength of optimal dispersal propensity. In fact, in shallow gradients the total dispersal is almost random even if $\mathrm{A}_i$ takes large values—letting $\nabla _x\mathrm{Q}\rightarrow 0$ in Equation (2) immediately implies that the optimal dispersal force vanishes to zero. It is also fairly intuitive to imagine that in shallow gradients individuals do not attain a sufficiently strong phenotype–environment match by moving along the gradient. In steep gradients, however, the backward movements induced by strong adaptive dispersal reduce the span of the region of sympatry, as described before.

Figure 4b shows that character displacement increases as the environmental gradient becomes steeper. In steeper gradients the maladaptive effects of random gene flow to range margins are stronger, and hence it enhances further the character displacement induced by interspecific competition. However, the rate of increase in character displacement as the gradient becomes steeper is much lower in the presence of adaptive dispersal. As we described before, in steep gradients matching habitat choice effectively pushes back the peripheral populations whose phenotypes are significantly displaced from the optimum.

When stabilizing natural selection is weak ($\mathrm{S}\rightarrow 0$), Figure 4c and d reveals a remarkable difference between the borders formed under matching habitat choice and those formed under random dispersal. When dispersal is only random and selection is sufficiently weak, no borders are formed between the species even though they express exceedingly strong character displacement. This is because the maladaptive effects of random gene flow are not “felt” by the species due to the weakness of the selection. Strong character displacements are expressed because only at such levels of displacement from trait optimum do the stabilizing effects of weak selection become sufficiently strong to balance the diversifying effects of competition. It is worth noting that the sharp increase in character displacement and span of the region of sympatry in Figure 4c and d, when $\mathrm{A}_1=\mathrm{A}_2=0$, arises when selection strength falls approximately below $0.05 \,\, \mathrm{Q}^{-2}/\mathtt {T}$. Although selection at this level is considered weak, it is still stronger than almost 25% of the estimates provided by Shirani and Miller (2022, Figure 1) based on the data available for 62 different species (Kingsolver et al., 2001, 2008). By contrast, range borders are formed under matching habitat choice even when selection is very weak. The region of sympatry expands only slightly with weak selection. The extent of character displacement is almost insensitive to selection strength and remains significantly reduced. The reason behind these distinct differences is that, as we discussed in previous section, maladaptive gene flow does not play a major role in coevolution of borders under matching habitat choice. Thus, the evolution of range limits in this case is rather insensitive to the strength of natural selection.

Another remarkable difference emerges for species with highly specialized individuals ($\mathrm{V}_i \rightarrow 0$, $i=1,2$). Figure 4e shows that with random-only dispersal, the length of the region of sympatry increases significantly when individuals become exceedingly specialized. This is because specialized populations effectively release themselves from competition. Hence, a broader region of sympatry is formed before the level of character displacement is sufficiently enhanced by maladaptive gene flow to halt range expansions. By contrast, with adaptive dispersal the length of the region of sympatry is fairly insensitive to changes in the degree of specialization. This is because matching habitat choice strongly controls the level of local trait variation (Figure 1f), keeping the specialized individuals sufficiently close together to still engage in strong competition. Figure 4f shows that the extents of character displacement are also remarkably different in different dispersal cases. With random-only dispersal, character displacement is fairly insensitive to the specialization level. In stark contrast, excessive specialization substantially reduces character displacement by dramatically strengthening the optimal dispersal force (see Equation (2)) and hence the backward movements it induces near range borders.

### Competitive advantages of matching habitat choice in steady and rapidly fluctuating environments

**Figure 5.**
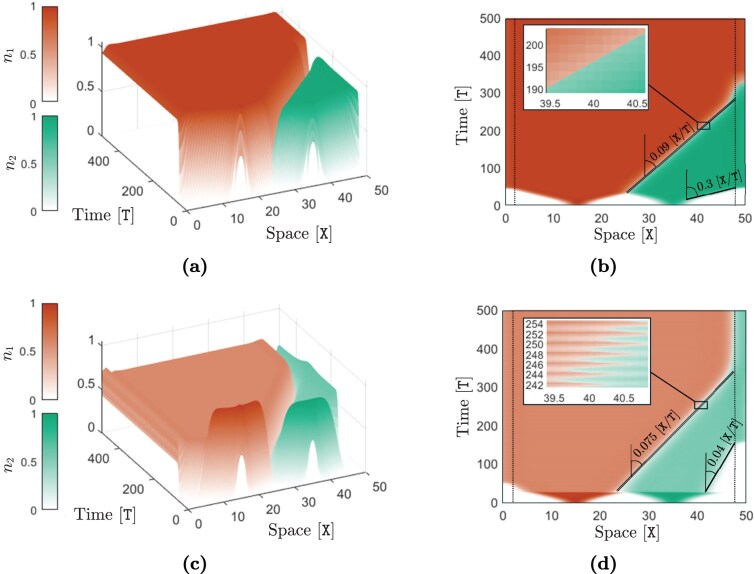
Competitive advantage of matching habitat choice in steady and rapidly fluctuating environments. The first species expresses a moderate level of phenotype-optimal dispersal with $\mathrm{A}_1 = 2 \,\, \mathtt {X}^2/\mathtt {T}$ whereas the second species disperses only randomly, $\mathrm{A}_2 = 0 \,\, \mathtt {X}^2/\mathtt {T}$. The environmental gradient is steep, $\nabla _x\mathrm{Q}= 1.5 \,\, \mathtt {Q}/\mathtt {X}$, and the rest of model parameters take their default values given in Supplementary Table S1. Graphs (a) and (b) in the upper panel show the results of a simulation with a steady (temporally constant) environment, whereas graphs (c) and (d) in the lower panel are associated with a periodically fluctuating environment. The evolution of the species’ population density $n_i$, $i=1,2$, is shown in each graph. Unlike Figure 1, the time axis is shown explicitly to allow for clear visualization of the moving borders. Graphs (b) and (d) show the top view of the same graphs shown in panels (a) and (c), respectively. The slopes specified on graphs (b) and (d) give the speed of the moving borders formed between the species (double line) as well as the other moving edge of the second (competitively weaker) species. The dotted lines in panels (b) and (d) indicate the boundaries of the region $\Omega _{\delta }$ as described in the discussion following Supplementary Equation (S14). It is assumed that outside $\Omega _{\delta }$ (near the habitat boundaries) the individuals change their optimal dispersal behavior to avoid crossing the habitat boundaries. In the lower panel, the temporal fluctuations in the environment start at $t = 30 \mathtt {T}$—when borders have effectively been established between the species—and follow a square waveform with period $2 \,\, \mathtt {T}$. Specifically, at the beginning of each period, the (linear) spatial profile of the trait optimum $\mathrm{Q}$ is abruptly shifted up by $4\,\, \mathtt {Q}$ and then remains fixed (at the shifted profile) for the first half of the period (for $1\,\, \mathtt {T}$). It is then shifted down by the same amplitude $4\,\, \mathtt {Q}$ back to the initial profile and remains fixed (at the initial profile) for the second half of the period (for $1\:\mathtt {T}$). This fluctuation pattern is repeated periodically until the end of the simulation.

We conclude our results by showing that, in steep environments, evolution of matching habitat choice in a species can give the species significant competitive advantage over a randomly dispersing similar species. The advantage is particularly notable for slowly growing (small $\mathrm{R}_i$) species in rapidly fluctuating environments, for example, species of montane birds living on temperate mountains with drastic seasonal variations in climate. We perform simulations of two species with identical parameters, but one dispersing through matching habitat choice with a moderate dispersal propensity $\mathrm{A}_1 = 2 \,\, \mathtt {X}^2/\mathtt {T}$, and the other dispersing only randomly. We consider both a steady environment (fixed $\mathrm{Q}$) and a periodically fluctuating environment. The results are shown in Figure 5.

The evolution of the species’ population density, shown for a steady environment in Figure 5a and b, confirms the competitive advantage of the species performing matching habitat choice. The borders formed between the species at the middle of the habitat are constantly pushed toward the randomly dispersing species, leading to exclusion of this species from the habitat. This competitive advantage of adaptive dispersal is particularly strong when the environment fluctuates rapidly and frequently. To see this, we simulate periodic abrupt fluctuations in the trait optimum, with period $2 \,\, \mathtt {T}$ and amplitude $4 \,\, \mathtt {Q}$. At the beginning of each period, the trait optimum $\mathrm{Q}$ is abruptly shifted up by $4\,\, \mathtt {Q}$ and then remains fixed for the first half of the period. It is then shifted down by $4\,\, \mathtt {Q}$ back to its initial value and remains fixed for the second half of the period. The results are shown in Figure 5c and d. We first note that frequent environmental fluctuations dramatically reduce range expansion speed of a randomly dispersing species. This is confirmed by comparing the speed $0.3 \,\, \mathtt {X}/\mathtt {T}$ of the freely moving edge of the randomly dispersing species in the steady environment (Figure 5b) with its speed of $0.04 \,\, \mathtt {X}/\mathtt {T}$ in the fluctuating environment (Figure 5d). By contrast, comparing the speeds $0.09 \,\, \mathtt {X}/\mathtt {T}$ and $0.075 \,\, \mathtt {X}/\mathtt {T}$ of the moving borders in the steady and fluctuating environments, respectively, indicates only a slight reduction due to the fluctuations. This further implies that the range expansion speed of the adaptively dispersing species is much less sensitive to environmental fluctuations. If the dispersal propensity of this species is made stronger, fluctuations are made more frequent, and habitat is made wider, then the randomly dispersing species may become extinct even before reaching the habitat boundary.

## Discussion

We find that predictions of models demonstrating the formation of range limits driven by interspecific competition are robust to assumptions about dispersal. Previous theory on competitively formed range limits (Case & Taper, 2000; Shirani & Miller, 2022) assumed that species disperse randomly, with the maladaptive effects of core-to-edge gene flow being a key contributor to the formation of range limits. This theory failed to address a situation where species disperse adaptively, which leads to peripheral populations receiving adaptive gene flow from core populations (Shirani & Miller, 2025). It thus seemed plausible that incorporating adaptive dispersal into models could lead to different outcomes on the possibility of range limits to be set by interspecific competition. However, this is not what we found. Instead, including matching habitat choice in models led to sharper range borders and reduced character displacement compared to models assuming random dispersal.

We showed that when species disperse adaptively, the key factor that contributes along with interspecific competition to formation of range limits—despite gene flow being adaptive—is the backward edge-to-core movement caused by adaptive dispersal at species’ range expansion wavefronts. The individuals which move randomly to expanding range margins, for example to explore new habitats or avoid kin competition, often face environments less suited to their phenotype. Following their preference for a matching habitat, they then move back toward the range core. When two competing species meet, these backward edge-to-core movements act to reduce the extent of character displacement induced by interspecific competition over the region of sympatry between the species. At equilibrium, the backward movements effectively cancel out the effects of random core-to-edge movements, resulting in almost no density change due to overall movements of individuals at and behind range borders. Interspecific competition, intensified further by reduced character displacement, then acts to sharply reduce species’ population density and set range limits.

The effects of matching habitat choice on increasing the sharpness of the borders and reducing character displacement become remarkably pronounced when stabilizing natural selection is weak or when individuals have a narrow niche breadth; see Figure 4c–f and Boxes 1 and 2. When dispersal is random, no borders may form in a linearly changing environment if the environment is weakly selective. That is, the species may become sympatric all over the habitat. Under matching habitat choice, by contrast, range borders are formed and remain relatively sharp, and species express reduced character displacement regardless of the strength of natural selection. When individuals’ niche breadth is narrow, that is, when individuals are highly specialized in utilizing resources or are very sensitive to environmental conditions, matching habitat choice dramatically reduces character displacement. However, we should note that the effects of matching habitat choice are prominent only if the environmental gradient is steep; see Figure 4a and b. In fact, considering its high cost (Bonte et al., 2012; Bowler & Benton, 2005; Clobert et al., 2009; Travis et al., 2012), matching habitat choice is unlikely to evolve as a dispersal strategy in shallow gradients (Shirani & Miller, 2025).

Matching habitat choice is hard to detect in nature and empirical evidence for its presence in nature is still limited (Camacho et al., 2020; Edelaar & Bolnick, 2012, 2019; Edelaar et al., 2023; Shirani & Miller, 2025). Our results suggest that the distinctive characteristics of range borders in the absence of sufficiently strong natural selection—that is, strikingly lower degrees of character displacement and sharper range borders—may be indications of matching habitat choice. The sharpness of the borders should be measured in our general choice of the unit of space (see Supplementary Section S1.3) and compared with species of similar characteristic parameters in equally steep gradients. This adds to the set of other hallmarks of matching habitat choice, such as adaptive gene flow to range margins and substantially reduced trait variation at central populations (Shirani & Miller, 2025). Our results further confirm the particular advantages of matching habitat choice for slowly growing (small values of $\mathrm{R}_i$) species in rapidly fluctuating environments, through enhancing the species’ competitive strength, invasion capacity, and persistence; see Figure 5.

In summary, we find that interspecific competition forms range limits across a range of assumptions about individual dispersal. That a variety of theoretical approaches generate the same general outcome (Case & Taper, 2000; Case et al., 2005; Goldberg & Lande, 2006; Price & Kirkpatrick, 2009; Roughgarden, 1979; Shirani & Miller, 2022; Tilman, 1982) is consistent with the perspective that interspecific competition can be a strong force setting range limits in nature. Dispersal behavior remains poorly understood in nature, and our theoretical findings point to rewarding lines of enquiry. Experiments measuring movements of individuals near range borders, adaptive or maladaptive effects of gene flow to peripheral populations, and phenotype-dependent competitive interactions would be useful to test the predictions of our work. In addition, observational data could be used to verify the result we present from theory, that character displacement is more common when dispersal is random compared to when dispersal is adaptive. Future empirical work can thus test whether and how our findings about the role of dispersal behavior in shaping species’ range limits apply in nature.

Matching habitat choice is a phenotype–environment matching dispersal strategy (Box 1). In the family of models such as the one we used in our study, the environment is modeled through the optimum value that it imposes on individuals’ phenotype. This trait optimum is often assumed to be determined by abiotic factors. However, a habitat can be described by biotic factors as well. In particular, it can be argued that phenotype-dependent competition (as in our model) can be included into the description of the environment. In this case, competition will directly contribute to individuals’ dispersal decision and direction, rather than the indirect contribution through creating a diversifying selection and inducing character displacement as in our study. Noting the conflicting effects of phenotype-(abiotic) environment matching dispersal and phenotype-dependent competition (Box 1), the individuals’ dispersal behavior in such a generalized form of matching habitat choice would then require assessment of a trade-off between avoiding competition and matching the optimum phenotype. Such an assessment could depend on population density as well. Including all such factors in the dispersal strategy makes it a rather fitness-maximizing (ideal free) optimal strategy, understanding the effects of which on coevolution of range borders can be an interesting direction of future research. Based on our analyses, we hypothesize that the formation of range borders by interspecific competition will still be robust to such an optimal dispersal behavior. However, the interactions between random and optimal dispersal and character displacement will likely converge to a different equilibrium state. An extension of our model that incorporates such a generalized habitat choice mechanism can help test our hypothesis and identify the impacts of optimal dispersal on the sharpness of the borders and extent of character displacement.

Finally, it is worth noting that adaptation to environments can also occur through other processes such as phenotypic plasticity and environment adjustment (niche construction). Understanding how these processes evolve independently or together, or along with matching habitat choice, and how the operation of each or a combination of them affects the coevolution of species’ range borders, is an important and challenging topic of research. The preliminary model-based studies by Edelaar et al. (2017), Scheiner et al. (2022), and Goldberg and Price (2022), for example, can help future efforts.

## Supplementary Material

qrag022_Supplemental_File

## Data Availability

This article does not use any data. The parameter values used to perform the computational studies are provided in the main text and the supplementary file. The MATLAB code developed for performing the computational studies is available on: https://github.com/Farshad-Shirani/2026_EvolutionLetters_FS_JRM_BGF.
