# Supplementary material for: Coevolution of species’ range borders: Interactions between interspecific competition, gene flow, and matching habitat choice
Source: Evol Lett. 2026 Jun 5;10(4):427–40. doi: 10.1093/evlett/qrag022 (PMC13451777; doi:10.1093/evlett/qrag022)
Supplement: Supplementary file 1 [file qrag022_supplemental_file.pdf]

Coevolution of Species' Range Borders: Interactions Between Interspecific Competition, Gene Flow, and Matching Habitat Choice

(Supplementary Material)

Farshad Shirani* Judith R. Miller† Benjamin G. Freeman‡

Contents

S1 Model Description **2**
S1.1 Historical background of the development of the model . . . 2
S1.2 Model equations . . . 3
S1.3 Model parameters: definitions, units, and ranges of values . . . 7
S1.4 Model assumptions . . . 8
S1.5 Model components and the underlying eco-evolutionary processes . . . 9
S1.6 Notational differences with preceding models . . . 13

S2 Numerical Computations **14**
S2.1 Numerical methods . . . 14
S2.2 Simulation initialization . . . 14
S2.3 Computing the contribution of dispersal components, selection, and competition to rate of changes in trait mean and population density . . . 15

S3 Range Evolution of a Solitary Species **15**
S3.1 Range evolution under random dispersal . . . 15
S3.2 Range evolution in the presence of matching habitat choice . . . 16

S4 Supplementary Figures **18**

*Department of Physics, Emory University, Atlanta, GA 30322, USA
†Department of Mathematics and Statistics, Georgetown University, Washington, DC 20057, USA
‡School of Biological Sciences, Georgia Institute of Technology, Atlanta, GA 30332, USA

S1. Model Description

Our theoretical study in the present work is based on a comprehensive mathematical model which we build as an immediate extension to a sequence of previously developed evolutionary models. The extension we perform does not require additional mathematical derivations and is accomplished by combining the results developed by Shirani and Miller (2022, 2025) and Shirani and Freeman (2025). Therefore, below we first briefly state some historical remarks on the development of the model and then provide its main equations, which are the equations we numerically solve to obtain the computational results of our work. To better understand the major eco-evolutionary processes that are incorporated into the model we then describe each of the underlying components of the model. We also specify the key assumptions that are used in building each component and deriving the final equations. We refer the reader to the work of Shirani and Miller (2022, 2025) and Shirani and Freeman (2025) for further details. We note that the notations we use to present the model are the same as those used by Shirani and Miller (2022, 2025). These notations differ from the notations used by the proceeding models, to some extent. These notational differences are listed in Table 2 in Section S1.6 below.

S1.1 Historical background of the development of the model

The mathematical model we use in our study has evolved through a sequence of developments, starting form the model proposed by Pease et al. (1989). In a seminal work aimed to test the "genetic swamping" hypothesis (Haldane, 1956; Mayr, 1963) as a cause of species' range limits, Kirkpatrick and Barton (1997) used the preliminary results of Pease et al. (1989) to develop a model of a species' range evolution over an environmental gradient in a fitness-related trait optimum. Using a system of partial differential equations, Kirkpatrick and Barton's model represents the joint evolution of the population density and the mean value of a quantitative phenotypic trait for a single (solitary) species in a one-dimensional geographic space. The species' dispersal in Kirkpatrick and Barton's model is assumed to be in the form of diffusive (random) migrations, and the population growth rate is assumed to be logistic. The species' adaptation to new environments is through the force of natural selection, which penalizes phenotypes that differ from the environment's optimum phenotype. Importantly, the trait variance in Kirkpatrick and Barton's model is assumed to remain constant in space and time. This unrealistic assumption was then relaxed by Barton (2001), who extended the model to further allow for the evolution of the trait variance.

The next important contribution to the development of models was made by Case and Taper (2000). In their influential work, Case and Taper extended the single-species model of Kirkpatrick and Barton to a community of competitively interacting species, where the competition between the individuals depended on their phenotype. However, trait variance in Case and Taper's model was still assumed to be constant in space and time; an assumption they called "tenuous". This assumption was then relaxed by Shirani and Miller (2022), who extended Case and Taper's model to higher dimensional geographic spaces and allowed for the evolution of trait variance jointly with the population density and trait mean of each species within the community. Shirani and Freeman (2025) subsequently added Allee effects to the model, which allowed for removing some biologically unrealistic solutions of the model that would arise in certain evolutionary regimes.

The individuals' dispersal (migration) in all the models described above is assumed to be ran-

dom. In a recent development aimed to study the effects of matching habitat choice on range evolution of a species, Shirani and Miller (2025) worked on a single-species version of their model and additionally incorporated phenotype-optimal dispersal, a special type of matching habitat choice in which individuals follow the direction of environmental gradient in the trait optimum to settle in the habitat best suited for their phenotype. In the present work, we further extend the single-species model developed by Shirani and Miller (2025) to a community of competitively interacting species with phenotype-optimal dispersal. We also incorporate Allee effects. Since each individual's optimal dispersal in Shirani and Miller's model depends only on its own phenotype and not on other individuals' phenotypes, the extension appears to be immediate and does not require further mathematical derivations. In Section S1.2 below, we provide the final equations of the model obtained by combining the equations developed by Shirani and Miller (2022, 2025) and Shirani and Freeman (2025). Since the presentation of the model with the community structure naturally appears to be complicated, an intuitive interpretation of each and every term in the equations is hard to establish. For the single-species case, the detailed interpretations of each terms in the model are available in Section 3 of the work by Shirani and Miller (2025). Such interpretations can provide helpful mechanistic insight into the key eco-evolutionary processes that shape the range dynamics predicted by the model.

S1.2 Model equations

We specify the model for a community of N species, although throughout the present work we only focus on the coevolution of borders between two species (N = 2). The species' habitat is modeled by an open rectangle $\Omega \subset \mathbb{R}^{\mathrm{m}}$ in an m-dimensional geographic space, $\mathrm{m} \in \{1, 2, 3\}$. In the present work we always consider a one-dimensional habitat, $\mathrm{m} = 1$. The evolution time horizon is denoted by T, where T can take any positive values and is set to be sufficiently large based on the specifics of each simulation. Each individual within each species is represented by a quantitative phenotypic trait such as body size. The environment imposes an optimum value for this quantitative trait, denoted by Q, which we typically assume changes linearly with x over the habitat Ω.

At every geographic location $x = (x_1, \ldots, x_{\mathrm{m}}) \in \Omega$ and time $t \in [0, T]$, the population density of the ith species, $i \in \{1, \ldots \mathrm{N}\}$, is denoted by $n_i(x, t)$, the mean value of the trait within the ith species is denoted by $q_i(x, t)$, and the variance of the trait is denoted by $v_i(x, t)$. The coevolution of the species' ranges in the model is represented by a system of partial differential equations that govern the joint evolution of these three population quantities for each species. In the equations of the model given below, for brevity we define a vector $u := (n_1, q_1, v_1, \ldots, n_{\mathrm{N}}, q_{\mathrm{N}}, v_{\mathrm{N}})$ that contains all these state variables.

The formulations of the eco-evolutionary processes incorporated into the model, as described in Section S1.5 below, also include several parameters whose definitions and biologically plausible ranges of values are given in Table 1. Further details of these parameters and their units are provided in Section S1.3. Note that in Table 1, the parameter $\mathrm{D}_i(x)$ is an $\mathrm{m} \times \mathrm{m}$ matrix ($\mathrm{D}_i(x) \in \mathbb{R}^{\mathrm{m} \times \mathrm{m}}$), whereas the rest of the parameters are scalar-valued. Moreover, the parameters S, U, J_i, V_i, Π, and κ are assumed to be constant in space (independent of x), whereas D_i, A_i, K_i, R_i, and Q can be variable in space. All these model parameters may also vary in time, although their dependence on t is not explicitly shown in the equations (S1)–(S14) below.

To write the equations of the model, we denote the partial derivative with respect to t by ∂_t,

Table S1: Definition and default values of the parameters of the model (S1)–(S14); (Shirani and Freeman, 2025; Shirani and Miller, 2022, 2025). The default values are used in all simulations presented in this work, unless otherwise is stated.

Parameter	Definition	Range	Default	Unit
m	Spatial dimension of the geographic space	$\{1,2,3\}$	1	—
N	Number of species	$\mathbb{N}$	2	—
$\mathrm{D}_i(x)$	Diffusion coefficient of the ith species' dispersal	$[0,25]^*$	1^*	$\mathtt{X}^2/\mathtt{T}$
$\mathrm{A}_i(x)$	Measure of the ith species' propensity to disperse optimally	$[0,10]$	4	$\mathtt{X}^2/\mathtt{T}$
$\mathrm{K}_i(x)$	Carrying capacity of the environment for ith species†	$(0,10]$	1	$\mathtt{N}/\mathtt{X}$
J_i	Critical population density (Allee threshold) of ith species	$[0,0.2]$	0.02	$\mathtt{N}/\mathtt{X}$
$\mathrm{R}_i(x)$	Maximum per capita growth rate of ith species	$[0.1,10]$	1	$1/\mathtt{T}$
V_i	Variance of individuals' phenotype utilization for ith species	$[0.25,25]$	4	$\mathtt{Q}^2$
κ	Asymmetric impact factor of phenotypic competitions	$[0,1]$	0	$1/\mathtt{Q}$
S	Measure of the strength of natural selection	$[0,2]$	0.2	$\mathtt{Q}^{-2}/\mathtt{T}$
U	Rate of increase in trait variance due to mutation	$[0,0.2]$	0.02	$\mathtt{Q}^2/\mathtt{T}$
$\mathrm{Q}(x)$	Optimum trait value for the environment	$[0,\infty)$	Linear‡	$\mathtt{Q}$
$\Vert\nabla_x \mathrm{Q}(x)\Vert_{\mathbb{R}^\mathrm{m}}$	Magnitude (steepness) of the gradient of the optimum trait	$[0,10]$	0.2	$\mathtt{Q}/\mathtt{X}$
Π	Maximum perceived value of the steepness of the optimum gradient	$[0,10]$	1	$\mathtt{Q}/\mathtt{X}$

*When $\mathrm{m}>1$, the range of values specified for $\mathrm{D}_i(x)$ can be considered for each entry of $\mathrm{D}_i(x)\subset\mathbb{R}^{\mathrm{m}\times\mathrm{m}}$. Typically, D_i is assumed to be diagonal.

†The carrying capacity K_i is defined as the maximum population density when individuals are completely generalist, that is, when $\mathrm{V}_i\to\infty$. When V_i is not large, the resulting competitive release allows for the maximum population density to exceed the value of K_i specified here.

‡The default value "Linear" specified for Q means that Q is typically considered to be a linear function of x over Ω.

the gradient with respect to x by ∇_x, the divergence with respect to x by div, the standard inner product in $\mathbb{R}^\mathrm{m}$ by $\langle\cdot,\cdot\rangle_{\mathbb{R}^\mathrm{m}}$, and the Euclidean norm in $\mathbb{R}^\mathrm{m}$ by $\Vert\cdot\Vert_{\mathbb{R}^\mathrm{m}}$.

We start by writing the equation for the evolution of the population density n_i of the ith species, $i=1,\dots,\mathrm{N}$, as

$$\partial_t n_i(x,t) = \mathrm{div}(\mathrm{D}_i(x)\nabla_x n_i(x,t)) \tag{S1a}$$

$$- \mathrm{div}\left(\mathrm{A}_i(x) n_i(x,t)\frac{q_i(x,t)-\mathrm{Q}(x)}{\mathrm{V}_i}\widetilde{\nabla_x\mathrm{Q}}(x)\right) \tag{S1b}$$

$$+ G_i(x,u(x,t))n_i(x,t). \tag{S1c}$$

where $G_i(x,u)$ denotes the mean growth rate of the population given by equation (S4) below, and $\widetilde{\nabla_x\mathrm{Q}}(x)$ denotes individuals' perceived environmental gradient (Section S1.5) given by equation (S14) below.

Likewise, the equation for the evolution of the trait mean q_i within the ith species is given as

$$
\begin{aligned}
\partial_t q_i(x,t) &= \operatorname{div}(\mathrm{D}_i(x)\nabla_x q_i(x,t)) && \text{(S2a)}\\
&+ 2\Big\langle \nabla_x \log n_i(x,t)\,,\, \mathrm{D}_i(x)\nabla_x q_i(x,t)\Big\rangle_{\mathbb{R}^\mathrm{m}} && \text{(S2b)}\\
&- \operatorname{div}\left(\mathrm{A}_i(x)\frac{v_i(x,t)}{\mathrm{V}_i}\widetilde{\nabla_x \mathrm{Q}}(x)\right) && \text{(S2c)}\\
&- \left\langle \nabla_x \log n_i(x,t)\,,\, \mathrm{A}_i(x)\frac{v_i(x,t)}{\mathrm{V}_i}\widetilde{\nabla_x \mathrm{Q}}(x)\right\rangle_{\mathbb{R}^\mathrm{m}} && \text{(S2d)}\\
&- \left\langle \nabla_x q_i(x,t)\,,\, \mathrm{A}_i(x)\frac{q_i(x,t)-\mathrm{Q}(x)}{\mathrm{V}_i}\widetilde{\nabla_x \mathrm{Q}}(x)\right\rangle_{\mathbb{R}^\mathrm{m}} && \text{(S2e)}\\
&+ H_i(x,u(x,t)), && \text{(S2f)}
\end{aligned}
$$

where $H_i(x,u)$ is defined by equation (S5) below.

Finally, the equation for the evolution of the trait variance v_i within the ith species is

$$
\begin{aligned}
\partial_t v_i(x,t) &= \operatorname{div}(\mathrm{D}_i(x)\nabla_x v_i(x,t)) && \text{(S3a)}\\
&+ 2\Big\langle \nabla_x \log n_i(x,t)\,,\, \mathrm{D}_i(x)\nabla_x v_i(x,t)\Big\rangle_{\mathbb{R}^\mathrm{m}} && \text{(S3b)}\\
&+ 2\Big\langle \nabla_x q_i(x,t)\,,\, \mathrm{D}_i(x)\nabla_x q_i(x,t)\Big\rangle_{\mathbb{R}^\mathrm{m}} && \text{(S3c)}\\
&- 2\left\langle \nabla_x q_i(x,t)\,,\, \mathrm{A}_i(x)\frac{v_i(x,t)}{\mathrm{V}_i}\widetilde{\nabla_x \mathrm{Q}}(x)\right\rangle_{\mathbb{R}^\mathrm{m}} && \text{(S3d)}\\
&- \left\langle \nabla_x v_i(x,t)\,,\, \mathrm{A}_i(x)\frac{q_i(x,t)-\mathrm{Q}(x)}{\mathrm{V}_i}\widetilde{\nabla_x \mathrm{Q}}(x)\right\rangle_{\mathbb{R}^\mathrm{m}} && \text{(S3e)}\\
&+ W_i(x,u(x,t)). && \text{(S3f)}
\end{aligned}
$$

where $W_i(x,u)$ is defined by equation (S6) below.

The nonlinear mappings G_i, H_i, and W_i in (S1c), (S2f), and (S3f) are defined as

$$
G_i(x,u) = B_i(n_i)\left(\mathrm{R}_i(x) - \frac{\mathrm{R}_i(x)}{\mathrm{K}_i(x)}\sum_{j=1}^{\mathrm{N}} M_{ij}(u)C_{ij}(u)n_j\right) - \frac{\mathrm{S}}{2}\left(\left(q_i - \mathrm{Q}(x)\right)^2 + v_i\right), \tag{S4}
$$

$$
H_i(x,u) = \Big(B_i(n_i)\mathrm{R}_i(x) - G_i(x,u)\Big)q_i - B_i(n_i)\frac{\mathrm{R}_i(x)}{\mathrm{K}_i(x)}\sum_{j=1}^{\mathrm{N}} L_{ij}(u)M_{ij}(u)C_{ij}(u)n_j + E_i(x,u), \tag{S5}
$$

$$
W_i(x,u) = \Big(B_i(n_i)\mathrm{R}_i(x) - G_i(x,u)\Big)(v_i - q_i^2) - B_i(n_i)\frac{\mathrm{R}_i(x)}{\mathrm{K}_i(x)}\sum_{j=1}^{\mathrm{N}} P_{ij}(u)M_{ij}(u)C_{ij}(u)n_j + Y_i(x,u), \tag{S6}
$$

where, letting $\Lambda_{ij} := \sqrt{\mathrm{V}_i/\bar{\mathrm{V}}_{ij}}$ with $\bar{\mathrm{V}}_{ij} := \frac{1}{2}(\mathrm{V}_i + \mathrm{V}_j)$; $i, j \in \{1, \ldots, \mathrm{N}\}$,

$$B_i(n_i) := \mathrm{B}_i \left(\frac{1}{1 + \exp\left(-\frac{n_i - \mathrm{J}_i}{\sigma_i}\right)} - \frac{1}{2} \right), \tag{S7}$$

$$C_{ij}(u) := \sqrt{\frac{2\bar{\mathrm{V}}_{ij}}{v_i + v_j + 2\bar{\mathrm{V}}_{ij}}} \Lambda_{ij} \exp(\kappa^2 \bar{\mathrm{V}}_{ij}), \tag{S8}$$

$$M_{ij}(u) := \exp\left(-\frac{(q_i - q_j + 2\kappa \bar{\mathrm{V}}_{ij})^2}{2(v_i + v_j + 2\bar{\mathrm{V}}_{ij})} \right), \tag{S9}$$

$$L_{ij}(u) := \frac{v_i(q_j - 2\kappa \bar{\mathrm{V}}_{ij}) + (v_j + 2\bar{\mathrm{V}}_{ij}) q_i}{v_i + v_j + 2\bar{\mathrm{V}}_{ij}}, \tag{S10}$$

$$P_{ij}(u) := \frac{v_i(v_j + 2\bar{\mathrm{V}}_{ij})}{v_i + v_j + 2\bar{\mathrm{V}}_{ij}} + L_{ij}(u)(L_{ij}(u) - 2q_i), \tag{S11}$$

$$E_i(x, u) := \frac{\mathrm{S}}{2} \left(2\mathrm{Q}(x) v_i + 2\mathrm{Q}(x) q_i^2 - \mathrm{Q}^2(x) q_i - 3 v_i q_i - q_i^3 \right), \tag{S12}$$

$$Y_i(x, u) := \frac{\mathrm{S}}{2} \left(2\mathrm{Q}(x) v_i q_i - 2\mathrm{Q}(x) q_i^3 - \mathrm{Q}^2(x)(v_i - q_i^2) - 3 v_i^2 + q_i^4 \right) + \mathrm{U}. \tag{S13}$$

The nonlinear function $B_i(n_i)$ given by (S7) adds Allee effects to the intrinsic population growth of the species, as described in Section S1.5 below. The key parameter in the formulation of $B(n_i)$ is the Allee threshold J_i as defined in Table 1. The parameter σ_i in (S7) determines how sharply the intrinsic growth rates increase to their maximum. The parameter B_i is adjusted such that the maximum value of $B_i(n_i)$ becomes equal to 1, so that the maximum intrinsic growth rate of the species becomes equal to R_i. For both species throughout our study we set $\mathrm{J}_i = 0.02$ `N/X` and $\sigma_i = 0.05$ `N/X`. The parameter B_i is then calculated based on theses values of J_i and σ_i. We obtain $\mathrm{B}_i = 2.6$. See also Figure S1 in the work by Shirani and Freeman (2025).

Finally, the individuals' perceived environmental gradient $\widetilde{\nabla_x \mathrm{Q}}(x)$ in (S2) and (S3) is defined as

$$\widetilde{\nabla_x \mathrm{Q}}(x) := \frac{\Pi}{\Pi + \|\nabla_x \mathrm{Q}(x)\|_{\mathbb{R}^{\mathrm{m}}}} \nabla_x \mathrm{Q}(x), \qquad x \in \Omega_\delta, \tag{S14}$$

where Π is a constant that determines the maximum steepness of the environmental gradient that is perceived by the individuals, as defined in Table 1; see also Section S1.5 below. The smaller habitat Ω_δ specified in (S14) includes all points of Ω except those that are closer than a constant δ to the boundary of Ω. This is due to some technical reasons described by Shirani and Miller (2025). It is assumed that the individuals of the species are able to perceive the boundary of the habitat once they become sufficiently close to it (closer than the constant δ), and they avoid crossing the boundary. To compute the solutions of the model, the definition of $\widetilde{\nabla_x \mathrm{Q}}$ in (S14) must be extended to the entire habitat Ω. The formulation for such an extension in the general case of an m-dimensional habitat is given in Appendix A of the work by Shirani and Miller (2025). For the one-dimensional habitat $\Omega = (a, b)$ that we consider in the present work, the extension is performed as follows:

$$\widetilde{\nabla_x \mathrm{Q}}(x) := \frac{\Pi}{\Pi + \|\nabla_x \mathrm{Q}(x)\|_{\mathbb{R}}} \Big(\nabla_x \mathrm{Q}(x) - \chi_\delta(x - a) \mathrm{d}_x^+ \mathrm{Q}(a) - \chi_\delta(x - b) \mathrm{d}_x^- \mathrm{Q}(b) \Big), \qquad x \in \Omega, \tag{S15}$$

where $\mathrm{d}_x^+ \mathrm{Q}(z)$ and $\mathrm{d}_x^- \mathrm{Q}(z)$ denote, respectively, the right-hand and left-hand derivatives of Q with respect to x evaluated at a point z, and

$$\chi_\delta(y) := \begin{cases} \exp\left(\frac{y^2}{y^2-\delta^2}\right), & |y| < \delta \\ 0, & |y| \geq \delta \end{cases} \tag{S16}$$

is a smooth cut-off function. We set the value of the parameter δ equal to $2\,\mathtt{X}$.

Solving the equations of the model (S1)–(S14) also requires setting appropriate boundary conditions at habitat boundaries. In general, different boundary conditions such as reflecting, periodic, absorbing, or a combination of these conditions can be set based on the context of the problem under study and dimension of the geographic space. Some technical considerations for setting the boundary conditions are given in Remark 1 and Appendix A.5 of the work by Shirani and Miller (2022). For the one-dimensional habitat $\Omega = (a, b)$ that we consider in the present work, we assume no phenotype flux through the habitat boundary which, as discussed by Shirani and Miller (2025: Section 2.4), results in the homogeneous Neumann boundary conditions

$$\nabla_x n_i = 0, \quad \nabla_x q_i = 0, \quad \nabla_x v_i = 0, \quad i = 1, \ldots \mathrm{N}, \qquad \text{on } \{a, b\} \times [0, T]. \tag{S17}$$

These conditions further imply that the habitat boundaries are reflecting. For example, if we conceptualize the habit to be representing the living environment of species of montane birds over an elevation-dependent temperature gradient, then the reflecting boundary condition at the high-elevation side of the habitat is equivalent to symmetrically (reflectively) expanding the habitat at the mountain top (Shirani and Freeman, 2025).

S1.3 Model parameters: definitions, units, and ranges of values

Due to the complexity of the equations of the model (S1)–(S14), analytical studies of the behavior of the model are rather impractical. Numerical analyses can result in biologically meaningful predictions provided that they are performed with carefully chosen biologically plausible parameter values. This further requires deliberate choices of units for the demographic, ecological, and evolutionary quantities present in the model. An extensive discussion has been provided by Shirani and Miller (2022: Section 3) on reasonable choices of units that give sufficient generality for the model, as well as plausible ranges of values that the parameters can take based on such units. The discussion has been extended by Shirani and Miller (2025: Section 2.5) to further include the parameters of phenotype-optimal dispersal. Here, we only describe the choices of units as needed for understanding the values given in Table 1. We also provide some comments on the values of a key parameter of the model: the steepness of the gradient in environmental trait optimum.

To specify units for the physical quantities of the model, one of the species in the community is first chosen as a *representative species*, for example, the one which is best adapted to the environment or has the widest niche. The units are then specified based on measurements of basic quantities within the representative species. Specifically, the unit of time, denoted by $\mathtt{T}$, is set to be equal to the mean generation time of the representative species. For the one-dimensional habitat that we consider in the present work, the unit of space, denoted by $\mathtt{X}$, is chosen such that the diffusion coefficient D of the representative population becomes unity. That is, we set $1\,\mathtt{X}$ to be equal to the root mean square of the dispersal distance of the population in $1\,\mathtt{T}$, divided by

$\sqrt{2}$. Estimates of the random component of the species' dispersal can be obtained, for example, by measuring dispersal distance of a subpopulation of individuals that are well adapted to the environment at the core of the population and hence do not perceive a significant force to disperse optimally. Having set the unit of space, the unit of measurement for population abundances, $\mathtt{N}$, is then chosen such that $1\,\mathtt{N}$ is equal to the carrying capacity of the environment for $1\,\mathtt{X}$ unit of habitat length (or, in general, $1\,\mathtt{X}^{\mathrm{m}}$ unit of habitat volume). Note that this results in the default value of the carrying capacity for the representative population becoming unity. Finally, letting $\mathtt{Q}$ denote the unit of measurement for the quantitative trait, we set $1\,\mathtt{Q}$ to be equal to one standard deviation of the trait values at the core of the representative population. A toy example is provided by Shirani and Freeman (2025) to describe how empirical measurements based on other choices of units can be converted to estimates based on the units we described above.

The steepness of environmental gradients in trait optima is a key factor in the evolution of phenotype-dependent dispersal strategies such as matching habitat choice (Shirani and Miller, 2025), as well as the contribution of gene flow to the coevolution of species' borders. Nevertheless, estimates of this key parameter are hard to obtain, and the available estimates are often based on different choices of units which make steepness comparisons almost impossible. In addition to measurements of the optimum trait value at different geographic locations, estimates of the environmental gradient based on the choices of units described above further requires measurements of standard deviation of the trait values, the mean dispersal distance, and the mean generation time of the representative population. The discussion given by Shirani and Miller (2022: Sect. 3.2) is based on limited data, and suggests a plausible range of values for the steepness of the gradient to be approximately between $0\,\mathtt{Q}/\mathtt{X}$ and $2\,\mathtt{Q}/\mathtt{X}$. The values close to $10\,\mathtt{Q}/\mathtt{X}$, as listed in Table 1, can be used to model physical barriers. Finding more accurate estimates for a broad range of species—based on choices of units which provide sufficient generality for comparison purposes (such as the units described above)—will be an important contribution to future studies of species adaptation and range evolution in heterogeneous environments.

S1.4 Model assumptions

The derivation of the equations of the model (S1)–(S14) relies on the following assumptions. The mathematical formulation associated with each assumption are given in Section S1.5 below.

(i) Random dispersal of the individuals in each species is diffusive.

(ii) An individual's environmental *phenotypic potential energy* for phenotype-optimal dispersal is proportional to the square of the difference between its phenotype value and the environment's optimum phenotype value.

(iii) Nonlinear environmental selection for an optimal phenotype is stabilizing.

(iv) The frequency of phenotype values within the species is normally distributed at every occupied point in space and for all time.

(v) In the absence of selection, the intrinsic growth rate of the individuals follows a logistic growth with Allee effects and phenotype-dependent competition. The carrying capacity and maximum growth rate of the individuals are independent of their phenotype.

(vi) Environmental resources vary continuously along a resource axis, and the resource utilization of each individual within each species follows a normal distribution. The resource axis can be identified by the phenotype axis through a one-to-one mapping. The individuals' *phenotype utilization distribution*, obtained by mapping the resource utilization distributions, remains normal.

(vii) The strength of intraspecific competition between individuals is determined by the overlap between their phenotype utilization curves, and can be asymmetric.

(viii) The probability of mutational changes from one phenotype to another phenotype depends on the difference between the phenotypes, and is assumed to be normally distributed with zero mean and constant variance.

(ix) For simplicity of model derivation, phenotypic variability is assumed to be predominantly genetic, and genetic variation is assumed to be predominantly additive. That is, $\mathrm{H}^2 \approx \mathrm{h}^2 \approx 1$, where H^2 and h^2 denote broad- and narrow-sense heritability, respectively.

S1.5 Model components and the underlying eco-evolutionary processes

The equations of the model (S1)–(S14) are derived from a basic evolutionary equation that specifies, over a small interval of time, changes in population density of individuals with phenotype $p \in \mathbb{R}$ within each species. To present this equation, let $\phi_i(x,t,p)$ denote the relative frequency of phenotype value $p \in \mathbb{R}$ in the ith population, at a location $x \in \Omega$ and time $t \in [0,T]$. Then $n_i(x,t)\phi_i(x,t,p)$ gives the population density of individuals with phenotype p in the ith population. The model assumes that changes in $n_i(x,t)\phi_i(x,t,p)$ over a small time interval of length $\tau \to 0$ results from the contribution of four major factors, as:

$$
\begin{aligned}
n_i(x,t+\tau)\phi_i(x,t+\tau,p) &- n_i(x,t)\phi_i(x,t,p) \\
&= \tau \operatorname{div}\Big(\mathrm{D}_i(x)\nabla_x\big(n_i(x,t)\phi_i(x,t,p)\big)\Big) && \text{(S18a)}\\
&\quad - \tau \operatorname{div}\Big(\mathrm{A}_i(x)n_i(x,t)\phi_i(x,t,p)\big(-\nabla_x\theta_i(x,p)\big)\Big) && \text{(S18b)}\\
&\quad + \tau g_i(x,t,p)n_i(x,t)\phi_i(x,t,p) && \text{(S18c)}\\
&\quad + \tau n_i(x,t)\partial_t^{(\mathrm{M})}\phi_i(x,t,p), \qquad i=1,\ldots,\mathrm{N}. && \text{(S18d)}
\end{aligned}
$$

The term (S18a) represents diffusive (random) dispersal of individuals to and from neighboring locations. The term (S18b) represents directed (optimal) dispersal of individuals in the direction that gives them maximum environmental match. The individuals' propensity A_i and perceived force $-\nabla_x\theta_i$ in (S18b) are described below. The term (S18c) models the intrinsic population growth, where the intrinsic growth rate is denoted by $g_i(x,t,p)$ and is described below. Finally, the term (S18d) represents mutational changes in the relative frequency of p in the ith population, where the rate of mutational changes is denoted by $\partial_t^{(\mathrm{M})}\phi_i(x,t,p)$ and is described below.

Intrinsic growth rates: A key factor that differentiates the density of individuals with different phenotypes is their intrinsic growth rate, modeled as

$$g_i(x,t,p) := \mathrm{R}_i(x)\left(1 - \frac{1}{\mathrm{K}_i(x)}\sum_{j=1}^{\mathrm{N}} n_j(x,t)\int_{\mathbb{R}} \alpha_{ij}(p,p')\phi_j(x,t,p')\mathrm{d}p'\right) B_i(n_i(x,t)) \tag{S19a}$$

$$- \frac{\mathrm{S}}{2}(p - \mathrm{Q}(x))^2, \tag{S19b}$$

for the ith species in the community of N species. Parameter R_i denotes the maximum per capita growth rate of the species, K_i denotes the carrying capacity of the environment for the species when the species' individuals are completely generalist (see below), S denotes the strength of stabilizing selection, and $\mathrm{Q}(x)$ denotes the environment's optimal trait value at location x (see also Table 1). In the absence of the term $B_i(n_i)$, (S19a) models a logistic growth, wherein the maximum growth rate R_i of the ith population occurs when all population densities n_j, $j = 1, \ldots, \mathrm{N}$, go to zero. The carrying capacity and maximum growth rate of individuals are assumed to be independent of their phenotype (Assumption (v)). In the presence of $B_i(n_i)$, defined by (S7), the population experiences Allee effects, so that it has negative growth rate when its density is lower than the Allee threshold J_i. The convolution term in (S19a) models the effects of phenotypic competition between the individuals as described below. The term (S19b) incorporates the effects of directional and stabilizing natural selection on individuals with phenotype p. It penalizes phenotypes which are different from the environmental optimal value $\mathrm{Q}(x)$. This penalizing effect is stronger for larger values of S.

Competition kernels: The competition kernel $\alpha_{ij}(p,p')$ in (S19a) represents the strength of per capita effects of individuals with phenotype p' in the jth species on the frequency of individuals with phenotype p in the ith species. Using the MacArthur-Levins overlap formula between *phenotype utilization curves* of each species (Shirani and Miller, 2022: Appendix A.2), the competition kernel is given as

$$\alpha_{ij}(p,p') = \Lambda_{ij}\exp(\kappa^2\bar{\mathrm{V}}_{ij})\exp\left(-\frac{(p - p' + 2\kappa\bar{\mathrm{V}}_{ij})^2}{4\bar{\mathrm{V}}_{ij}}\right), \quad i,j = 1,\ldots,\mathrm{N}, \tag{S20}$$

where $\Lambda_{ij} := \sqrt{\mathrm{V}_i/\bar{\mathrm{V}}_{ij}}$ with $\bar{\mathrm{V}}_{ij} := \frac{1}{2}(\mathrm{V}_i + \mathrm{V}_j)$. Parameter κ is used to model asymmetry in phenotypic competitions. Throughout this work, however, we always assume symmetric competition between phenotypes, with $\kappa = 0$. Parameter V_i denotes the variance of *phenotype utilization distributions*, which constitute the phenotype utilization curves and are assumed to be normal,

$$\psi_{i,p}(\tilde{p}) = \frac{1}{\sqrt{2\pi\mathrm{V}_i}}\exp\left(-\frac{(\tilde{p} - p)^2}{2\mathrm{V}_i}\right), \quad i = 1,\ldots,\mathrm{N}. \tag{S21}$$

The phenotype utilization distribution $\psi_{i,p}$ of individuals with phenotype p in the ith population are obtained from their presumed normally distributed resource utilization distribution, after identification of the resource axis with the phenotype axis (Shirani and Miller, 2022: Appendix A.2). The distribution function $\psi_{i,p}(\tilde{p})$ can be interpreted as a function that gives the probability density that an individual with phenotype p in ith species will utilize a unit of resource that is expected to be mostly utilized by (is most favorable for) an individual with phenotype $\tilde{p}$. In trait-based

niche quantification frameworks (Ackerly and Cornwell, 2007; Violle and Jiang, 2009) the variance of phenotype utilization distributions can be used to quantify within-phenotype component of a species' niche breadth. Moreover, the resource-phenotype identification used to construct phenotype utilization distributions can be conceptualized to represent the empirical relationship between functional response traits and environments. This interpretation inspires the choice of the phenotypic potential energy function for optimal dispersal, as described below.

Carrying capacity: The phenotype-dependence of the competition in the model creates an ambiguity in the definition of K_i, which needs to be clarified (Shirani and Freeman, 2025). As stated above, K_i denotes the maximum population density of the ith species when the species' individuals are "completely generalist", that is, when $\mathrm{V}_i \to \infty$, $i = 1, \ldots \mathrm{N}$. This is because $\alpha_{ij}(p, p') \to 1$ in (S20) when $\mathrm{V}_{ij} \to \infty$, regardless of the values of p and p'. Setting $\alpha_{ij}(p, p') = 1$ in (S19) verifies that completely generalist species with density $n_i > \mathrm{K}_i$ will have negative intrinsic growth, hence the population density of such species will be bounded by K_i. However, when individuals are specialized in utilizing resources, they become partially released from competition, meaning that $\alpha_{ij}(p, p') < 1$. In this case, g_i can take positive values for some $n_i > \mathrm{K}_i$. That is, specialized species can grow to population densities greater than K_i; see, for example, the curves of range expansion wave amplitudes given by Shirani and Miller (2025: Fig. 2b). Therefore, even though K_i is phenotype-independent, the carrying capacity of species—defined generally as species' maximum population density—depends both on the distribution of phenotypes and individuals' phenotype-based resource utilization.

Changes due to mutation: The rate of mutational changes in the frequency of phenotypes in the ith population is modeled as

$$\partial_t^{(\mathrm{M})} \phi_i(x, t, p) := -\eta \phi_i(x, t, p) + \eta \int_{\mathbb{R}} \nu(p - p') \phi_i(x, t, p') \mathrm{d}p', \quad i = 1, \ldots, \mathrm{N}, \tag{S22}$$

where $\eta \geq 0$ is the mutation rate per capita per generation. Following Assumption (viii), the mutation kernel $\nu(\delta p)$ denotes the probability density that by mutation a phenotype p changes to a phenotype $p' = p + \delta p$. The first term in (S22) incorporates the reduction rate in the frequency of phenotype p within the ith species due to mutation to other phenotype values. The second term incorporates the growth rate in the frequency of phenotype p, due to mutations from other phenotype values to p.

Phenotype-optimal dispersal: The phenotype-optimal dispersal in the ith species is modeled by the advection term (S18b), which is analogous to the drift component of drift-diffusion models of particles flowing in a fluid. The *propensity* of the ith species' individuals to disperse optimally, denoted by A_i, is analogous to the *mobility* of the particles. The *phenotypic dispersal force* $-\nabla_x \theta_i(x, p)$ induced by individuals' perceived *phenotypic potential energy* $\theta_i(x, p)$ is analogous to the force acting on particles derived from an external potential energy. The following model is used for the phenotypic potential energy function of an individual with phenotype p in the ith species at a habitat location x,

$$\theta_i(x, p) := \frac{\left(p - \mathrm{Q}(x)\right)^2}{2\mathrm{V}_i}. \tag{S23}$$

This potential energy function is proportional to a first-order approximation of the phenotype utilization distribution $\psi_{i,p}(\mathrm{Q}(x))$. It can therefore be interpreted as how well or poorly an individual with phenotype p can utilize resources at its current location x, which are most favorable to individuals with optimum phenotype $\mathrm{Q}(x)$. If the individual's phenotype matches the optimum phenotype perfectly, then there is no phenotypically and environmentally induced force on the individual to disperse. If the individual's phenotype differs significantly from the environment's optimum—measured relative to the species' phenotype utilization variance V_i—then the individual perceives a high potential energy to disperse to habitat locations of better quality that match its phenotype. However, unless a sufficiently large gradient in the phenotypic potential energy is perceived by the individual, a high potential energy $\theta_i(x,p)$ does not generate a significant dispersal force $-\nabla_x\theta_i(x,p)$ on the individual. Such a gradient is present if the environmental gradient $\nabla_x\mathrm{Q}$ is sufficiently steep and the individual is sufficiently sensitive to it.

The phenotypic potential energy (S23) gives an optimal dispersal force term $-\nabla_x\theta_i$ as

$$-\nabla_x\theta_i(x,p) = \frac{p-\mathrm{Q}(x)}{\mathrm{V}_i}\,\nabla_x\mathrm{Q}(x). \tag{S24}$$

If the magnitude of the environmental gradient $\|\nabla_x\mathrm{Q}(x)\|_{\mathbb{R}^\mathrm{m}}$ is zero (where $\|.\|_{\mathbb{R}^\mathrm{m}}$ denotes the Euclidean norm in $\mathbb{R}^\mathrm{m}$) or is perceived as equal to zero due to insensitivity of an individual to the gradient, then the perceived directed dispersal force on the individual is zero—regardless of the presence of a phenotype-environment mismatch $p \neq \mathrm{Q}(x)$. In this case the individual only disperses randomly, due to the diffusion term (S18a). When $\|\nabla_x\mathrm{Q}(x)\|_{\mathbb{R}^\mathrm{m}} > 0$, an individual whose phenotype p does not perfectly match the optimum phenotype will perceive a force pushing it to disperse optimally. If $p > \mathrm{Q}(x)$ the optimal dispersal will be in the direction of the environmental gradient, and if $p < \mathrm{Q}(x)$, the optimal match will be in the direction opposite to the environmental gradient.

A biological organism may not develop a perception of the phenotypic dispersal force (S24) in its exact mathematical sense. However, to make the modeling mathematically feasible, the phenotypic potential $(p-\mathrm{Q})/\mathrm{V}_i$ part of the dispersal force is assumed to be perceived by the individuals exactly. However, a biologically more reasonable perception of the environmental gradient, denoted by $\widetilde{\nabla_x\mathrm{Q}}$ and formulated by (S14), is used for the gradient part. That is, the individual's *perceived dispersal force* for phenotype-optimal dispersal,

$$-\widetilde{\nabla_x\theta_i}(x,p) = \frac{p-\mathrm{Q}(x)}{\mathrm{V}_i}\,\widetilde{\nabla_x\mathrm{Q}}(x), \tag{S25}$$

is substituted into (S18b) for the dispersal force term. The constant Π in the formulation of the perceived gradient (S14) determines the maximum perceived magnitude of the gradient. When $\|\nabla_x\mathrm{Q}(x)\|_{\mathbb{R}^\mathrm{m}}$ is much smaller than Π, the perceived gradient approximately equals the actual gradient. When $\|\nabla_x\mathrm{Q}(x)\|_{\mathbb{R}^\mathrm{m}}$ is much larger than Π, the magnitude of the perceived gradient approximately saturates to the maximum value Π. The direction of the perceived gradient, however, will always be the same as the direction of the actual gradient. The perceived gradient $\widetilde{\nabla_x\mathrm{Q}}$ can be further interpreted, in some sense, as the sensitivity of the individuals' dispersal force to changes in habitat quality. See Remark 3 of the work by Shirani and Miller (2025) for further details.

Table S2: Notational comparisons between the model presented in this work and Case & Taper's model (2000). The presence of the enumeration index i in a variable implies that the variable can take a different value for each species.

**Parameter/variable/function description**	**Present work**	**Case & Taper's (2000)**
The random variable denoting phenotype values	p	z
Population density	n_i	N_i
Trait mean	q_i	$\bar{z}_i$
Trait variance	v_i	V_p
Per capita intrinsic growth rate	g_i	w_i
Mean intrinsic growth rate	G_i	$\bar{w}_i$
Competition kernel	α_{ij}	α
Relative frequency (distribution) of phenotypes	ϕ_i	p_i
Mutational changes in frequency of phenotypes	$\partial_t^{(\mathrm{M})}\phi_i$	—
Diffusion coefficient of species' dispersal	D_i	D
Measure of Species' propensity to disperse optimally	A_i	—
Carrying capacity of the environment	K_i	K
Critical population density (Allee threshold)	J_i	—
Maximum per capita growth rate	R_i	r_i
Variance of individuals' phenotype utilization	V_i	V_u
Asymmetric impact factor of phenotypic competitions	κ	κ
Measure of the strength of natural selection	S	$1/V_s$
Rate of increase in trait variance due to mutation	U	—
Optimal trait value for the environment	Q	θ

S1.6 Notational differences with preceding models

Compared with the notations used in the models of Kirkpatrick & Barton (1997) and Case & Taper (2000), some notational changes have been made here in presenting the equations and the underlying components of the model. These notational changes are made for several reasons, namely, to distinguish model variables from model parameters and mappings of model variables, to avoid conflicts with commonly used notations in mathematical analysis of PDEs, and to allow for further notational considerations in future extensions and analyses of the model. To facilitate comparisons with the preceding models, in Table 2 we provide the list of model parameters and variables with the notations used in the present work (as well as in Shirani and Freeman (2025); Shirani and Miller (2022, 2025)) and those used in Case and Taper (2000) (as well as in Kirkpatrick and Barton (1997) and other works in this family of models).

S2. Numerical Computations

S2.1 Numerical methods

The numerical method used in the work by Shirani and Miller (2022, 2025) is further extended in the present work to obtain the simulation results for the range evolution of two competing species with phenotype-optimal dispersal, and for quantifying the effects of directed gene flow on coevolution of the species' boundary. The method is based on an Alternating Direction Implicit (ADI) scheme with two stabilizing correction stages (Hundsdorfer, 2002). In each iteration of the scheme, instead of solving the nonlinear algebraic equations involved in the computations, the linearized version of these equations are solved. The linearization is performed by computing the associated Jacobian matrix of the system symbolically (by hand) so that the Jacobian matrix is exact. The iteration time steps are then made smaller to compensate for the linearization error. The first and second space derivatives are approximated using fourth-order centered differences. See Appendix B of the work by Shirani and Miller (2022) for further details.

In all simulations, we discretized the one-dimensional space (habitat) with a uniform mesh of size $\Delta x = 0.1\ \mathtt{X}$. For the majority of the simulations in which we aimed to compute the equilibrium curves accurately, we used small time steps of length $\Delta t = 0.002\ \mathtt{T}$. For simulations that did not require computation of fully accurate solutions, we chose longer time steps of $\Delta t = 0.005\ \mathtt{T}$ or $\Delta t = 0.01\ \mathtt{T}$.

The numerical computations of the solutions of the model are challenging, mainly due to the terms $\nabla_x \log n_i$ in the equations (S2) and (S3) of the model. These terms are undefined when the population densities n_i are zero. See further details in Section 4.1 of the work by Shirani and Miller (2022). Even if we initialize the populations such that at least an infinitesimal population density is initially present everywhere, there is still a chance of reaching numerical singularities if the density of a species undergoes a long density decline (or extinction) regime—especially in the presence of Allee effect. Similar to the approach taken by Shirani and Freeman (2025), we avoid this singularity problem by replacing the terms $\nabla_x \log n_i$ with $\nabla_x \log(n_i + \epsilon)$, $\epsilon > 0$. We set $\epsilon = 10^{-5}$, which works well in our simulations and does not result in any noticeable impact on the solutions compared with the solutions of the original equations.

S2.2 Simulation initialization

To obtain the numerical results presented here and in the main text, we consider a one-dimensional habitat $\Omega = (0\,\mathtt{X}, 50\,\mathtt{X}) \subset \mathbb{R}$ with the reflecting boundary conditions (S17). We initially introduce the two populations allopatrically, the 1st population centered at $c_1 = 15\ \mathtt{X}$ and the 2nd population centered at $c_2 = 25\ \mathtt{X}$. We set the initial population densities at $t = 0\ \mathtt{T}$ as $n_i(x, 0) = 0.5\varrho(|x - c_i|)$, $i = 1, 2$, where ϱ is a bump function. We assume that the initial populations are perfectly adapted to the environment at their center, $q_i(c_i, 0) = \mathrm{Q}(c_i)$, but poorly adapted at other habitat locations, $\nabla_x q_i(x, 0) = 0.5\nabla_x \mathrm{Q}(x)$ for all $x \in \Omega$. Finally, we set the initial populations' trait variance equal to $v_i(x, 0) = 1\ \mathtt{Q}^2$. The values of the model parameters used in each simulation are specified independently for each figure, based on the values given in Table 1.

S2.3 Computing the contribution of dispersal components, selection, and competition to rate of changes in trait mean and population density

The contribution of each component of dispersal to the rates of changes in species' trait mean, $\partial_t q_i$, and species' population density, $\partial_t n_i$, $i = 1, 2$ are shown in Figures 2 of the main text (for 1st species) and Figure S1 here (for 2nd species). The contribution of the local effects of selection and competition to $\partial_t q_i$ and $\partial_t n_i$ are also shown in Figure 3 of the main text and Figure S3 for 1st species. For computing the dispersal contributions we use the terms (S1a), (S1b), and (S2a)–(S2e) in the model, which directly originate from the random and optimal dispersal of phenotypes as in (S18a) and (S18b). For computing the local contributions due to selection and competition we use the terms (S1c) and (S2f) in the model, which originate from the intrinsic growth rate of the population of phenotypes as in (S18c). These terms, along with (S3f), control the local dynamics of the populations.

Specifically, the curves in Figure 2a are computed as the sum of the terms (S2a) and (S2b) in the equations of the model, with $i = 1$. The curves in Figure 2b are computed as the sum of the terms (S2c)–(S2e), with $i = 1$. The contribution of the total dispersal to $\partial_t q_1$, shown in Figure 2c is computed as the sum of the curves in Figures 2a and 2b, that is, the sum of the terms (S2a)–(S2e), with $i = 1$. The curves in Figures S1a–S1c are computed similarly, using the same terms in model equations, but with $i = 2$. The curves of local effects in Figures 3b and S3b are computed using (S2f) with $i = 1$. The final curves of $\partial_t q_1$ in Figures 3c are computed as the sum of the curves in Figures 3a and 3b. Similarly, the final curves of $\partial_t q_1$ in Figure S3c are computed as the sum of the curves in Figures S2c and S3b.

For contribution of the dispersal terms to $\partial_t n_1$, the curves in Figure 2d are computed using (S1a) with $i = 1$, the curves in Figure 2e are computed using (S1b), and the curves in Figure 2f are computed as the sum of (S1a) and (S1b). Similarly, the curves of contributions to $\partial_t n_2$, shown in Figures S1d–S1f are computed using the same terms in model equations, but with $i = 2$. The curves of local effects in Figures 3e and S3e are computed using (S1c) with $i = 1$. The final curves of $\partial_t n_1$ in Figures 3f are computed as the sum of the curves in Figures 3d and 3e. Similarly, the final curves of $\partial_t n_1$ in Figure S3f are computed as the sum of the curves in Figures S2f and S3e.

S3. Range Evolution of a Solitary Species

The range evolution of a solitary species using the reduction of our model to a single-species is extensively analyzed by Shirani and Miller (2022, 2025), as well as many of the preceding important works (Barton, 2001; Kirkpatrick and Barton, 1997; Polechová and Barton, 2015). Below we provide a short summary of the key results.

S3.1 Range evolution under random dispersal

With random-only dispersal, Kirkpatrick and Barton (1997) show that genetic swamping can cause formation of a limited range when the environment is sufficiently steep. Moreover, they show that the population becomes extinct if the steepness of the gradient exceeds a critical value that depends on the strength of selection, dispersal coefficient, and maximum growth rate. However, when trait variance is allowed to evolve, Barton (2001) and Shirani and Miller (2022) show that the limited

range regime predicted by Kirkpatrick and Barton (1997) in a linear environment does not exist. The population will either expand its range indefinitely or go extinct, depending on the steepness of the gradient. This is because, the trait variance is inflated by random gene flow as the steepness of the environment increases. As a results, the standing phenotypic load imposed by natural selection on the population growth rate increases with the steepness of the gradient. This leads to population extinction when the steepness of the gradient exceeds a critical value; see (Shirani and Miller, 2022: Sec. 4.2 and Fig. 4) and (Shirani and Miller, 2025: Fig. 2a). In the absence of Allee effect, the critical gradient steepness is given as

$$\|\nabla_x \mathrm{Q}\|_{\mathbb{R}}^{(\max)} = \sqrt{\frac{2\mathrm{R}^2}{\mathrm{SD}} - \frac{\mathrm{U}}{2\mathrm{D}}} \approx \sqrt{\frac{2\mathrm{R}^2}{\mathrm{SD}}}, \tag{S26}$$

where the enumeration index i is dropped from the parameters, as we discuss only a single species here. The approximation in (S26) is made based on the fact that, typically, $\mathrm{U} \ll 4\mathrm{R}^2/\mathrm{S}$. Note that (S26) implies that the critical gradient steepness decreases when R decreases or when S increases. This means that, slowly-growing species and species under strong selection are at higher risk of extinction at steep gradients. See (Shirani and Miller, 2022: Remark 4) and (Shirani and Miller, 2025: Remark 6) for more details.

The existence of the critical (extinction) gradient steepness described above also implies that steepening nonlinear gradients can limit species' range (Bridle et al., 2019; Polechová, 2018; Polechová and Barton, 2015). This follows from the fact that gradient is a mathematical notion that is understood (defined) locally. A steepening nonlinear environmental gradient can be approximated by a piecewise linear gradient, such that the steepness of linear pieces increases along the environment. It then follows immediately from the predictions of the models (in linear environments) that the local population density over the linear pieces decreases as the steepness of the pieces increases. If the steepness of the steepening gradient eventually exceeds the critical extinction threshold predicted by the models, the local populations over exceedingly steep pieces will fail to service. This results in the formation of a range limit by the steepening gradient. Figures 10a and 10b of the work by Shirani and Miller (2022), for example, show how species' range expansion stops at a steepening gradient.

S3.2 Range evolution in the presence of matching habitat choice

Similar to the random-only dispersal case, our model predicts that in the presence of phenotype-optimal dispersal, the species' range evolution in linear environments can have two different regimes: indefinite range expansion and complete extinction (Shirani and Miller, 2025). However, phenotype-optimal dispersal substantially increases the critical gradient steepness beyond which the species becomes extinct. This is because matching habitat choice substantially reduces local trait variance, and hence the phenotypic load imposed by selection on population growth (Shirani and Miller, 2025: Fig. 2a). Therefore, the species' chance of survival at steep gradients is significantly enhanced by dispersing through matching habitat choice. Moreover, steepening nonlinear gradients should reach a much higher level of steepness in order to limit the range expansion of a species that disperses through matching habitat choice. For the majority of species in nature, such a steep gradient may not be realistically present or may just correspond to obvious physical barriers or habitat discontinuities.

In the absence of Allee effects, the critical gradient steepness (extinction steepness) can be computed by solving

$$\|\nabla_x \mathrm{Q}\|_{\mathbb{R}}^{(\max)} = \sqrt{\frac{4\mathrm{R}^2 - \mathrm{SU}}{2\Big(\mathrm{SD} - 2\mathrm{R}(\mathrm{A}/\mathrm{V})\Upsilon\big(\, \|\nabla_x \mathrm{Q}\|_{\mathbb{R}}^{(\max)}\,\big)\Big)}}, \tag{S27}$$

where $\Upsilon\big(\, \|\nabla_x \mathrm{Q}\|_{\mathbb{R}}^{(\max)}\,\big) := \frac{\Pi}{\Pi + \|\nabla_x \mathrm{Q}\|_{\mathbb{R}}^{(\max)}}$. Similar to the random-only dispersal case, this critical steepness decreases as R decreases or as S increases. However, dispersing through matching habitat choice can still make the critical gradient steepness significantly greater than the gradients that realistically present in nature, even for (vulnerable) slowly-growing species under strong selection; see (Shirani and Miller, 2025: Fig. 3). This implies that, this class of vulnerable species may particularly benefit from evolving matching habitat choice, especially when the environment is fluctuating rapidly and frequently (Shirani and Miller, 2025: Figs. 6 and 7).

S4. Supplementary Figures

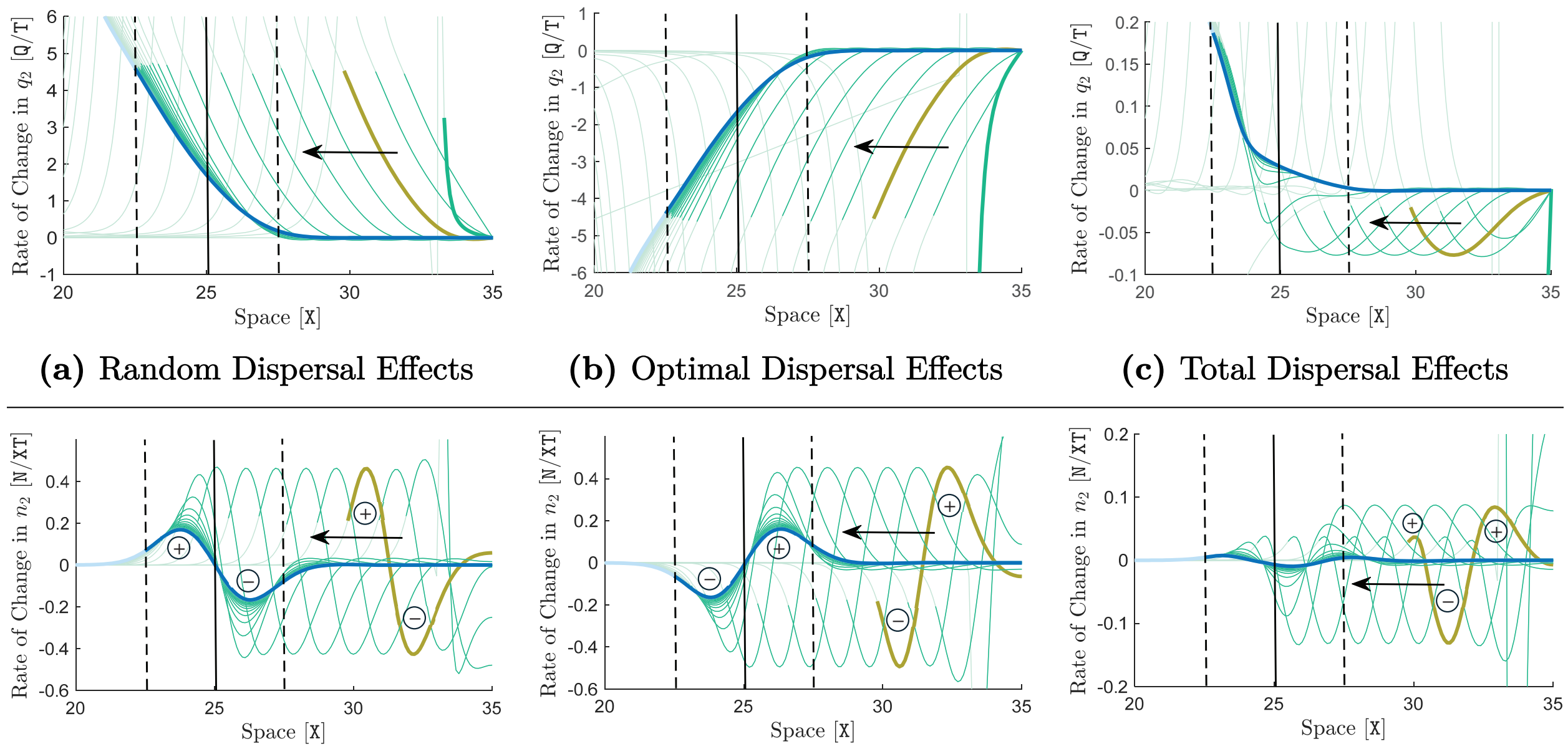

(a) Random Dispersal Effects
(b) Optimal Dispersal Effects
(c) Total Dispersal Effects

(d) Random Dispersal Effects
(e) Optimal Dispersal Effects
(f) Total Dispersal Effects

Figure S1: Effects of random and optimal dispersal on rates of changes in trait mean and population density. The results shown here are associated with the same analysis made to obtain the results shown in Figure 2 of the main text, but here we show the curves for the 2nd species. Specifically, the graphs are associated with the same simulation performed to obtain the results shown in the right panel of Figure 1, that is, with $\mathrm{A}_1 = \mathrm{A}_2 = 10\ \mathtt{X}^2/\mathtt{T}$ and $\nabla_x \mathrm{Q} = 1.5\ \mathtt{Q}/\mathtt{X}$. The contributions of random dispersal, phenotype-optimal dispersal, and total dispersal to the rate of change of trait mean within the 2nd species, $\partial_t q_2$, are shown in graphs (a)–(c) in the upper panel. The contributions of these dispersal components to the rate of change of population density of the 2nd species, $\partial_t n_2$, are shown in the lower panel. The details of the computations associated with these contributions are described in supplementary Section S3.3. The curves in all graphs are shown only for the left half of the species' range, where it meets and coevolves with the 1st species. In all graphs, curves are shown at every 4 $\mathtt{T}$, and the parts of the curves which lie outside the species' range ($n_1 < 0.02$) are made transparent. The final equilibrium curves obtained (approximately) at the end of the simulation ($T = 300\ \mathtt{T}$) are highlighted in blue. The sample curves highlighted in olive are associated with the species' range expansion regime before meeting the 1st species. The solid black lines indicate the center of the habitat where the interface between the two species (Figure 1b in the main text) is formed. The dashed lines indicate the boundaries of the region of sympatry formed at the interface.

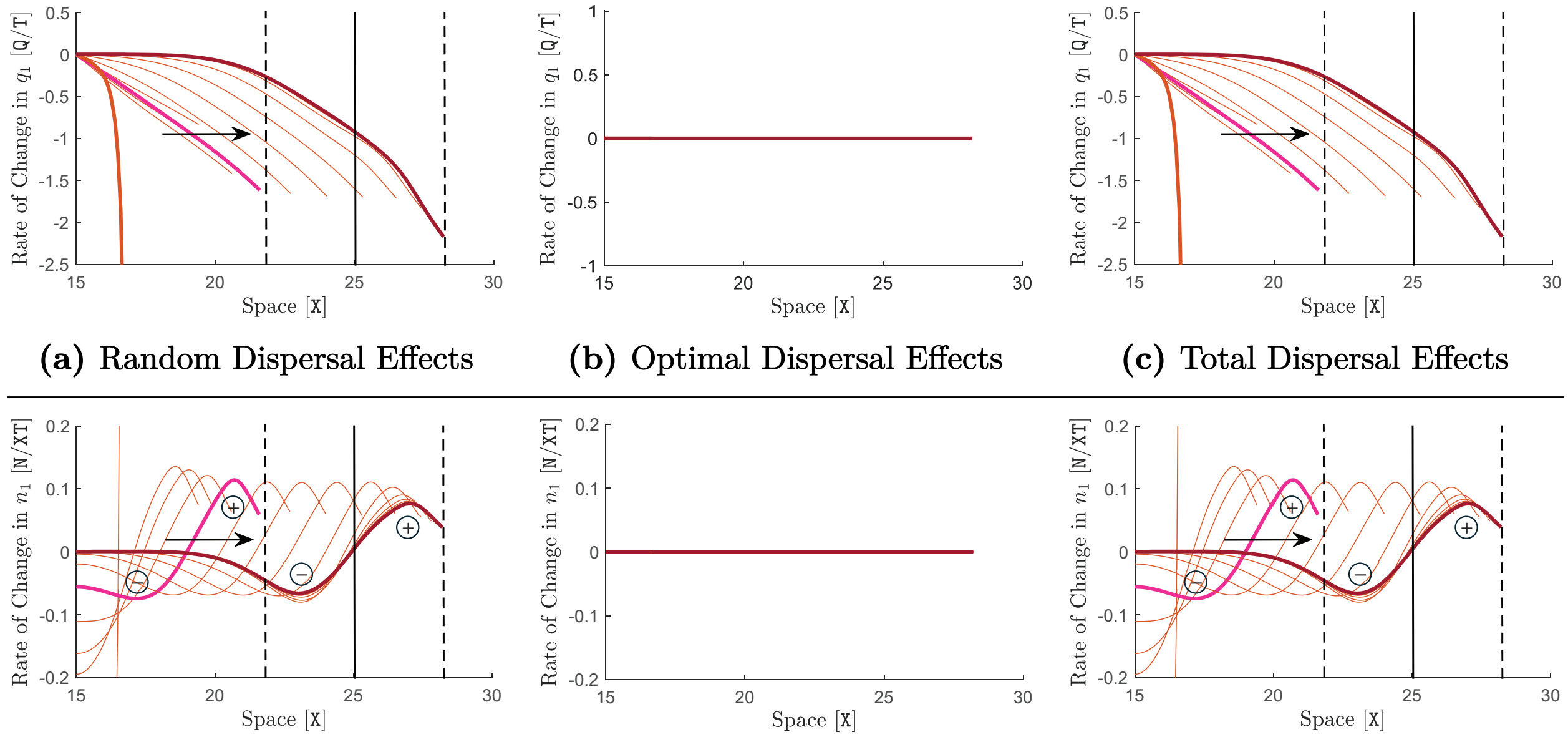

(a) Random Dispersal Effects **(b)** Optimal Dispersal Effects **(c)** Total Dispersal Effects

(d) Random Dispersal Effects **(e)** Optimal Dispersal Effects **(f)** Total Dispersal Effects

Figure S2: Effects of random-only dispersal on rates of change in trait mean and population density in the absence of phenotype-optimal dispersal. The results shown here are similar to those shown in Figure 2 of the main text, but here in the absence of phenotype-optimal dispersal (only with random dispersal). Specifically, the graphs are associated with the same simulation performed for the results shown in the left panel of Figure 1 in the main text, that is, with $\mathrm{A}_1 = \mathrm{A}_2 = 0\ \mathtt{X}^2/\mathtt{T}$ and $\nabla_x \mathrm{Q} = 1.5\ \mathtt{Q}/\mathtt{X}$. Since phenotype-optimal dispersal is absent, its contribution to $\partial_t q_1$ and $\partial_t n_1$ is zero. The total contribution of dispersal to $\partial_t q_1$ and $\partial_t n_1$ is then equal to the contribution of random dispersal. The same description as in Figure 2 of the main text holds for the curve colors and the solid and dashed lines.

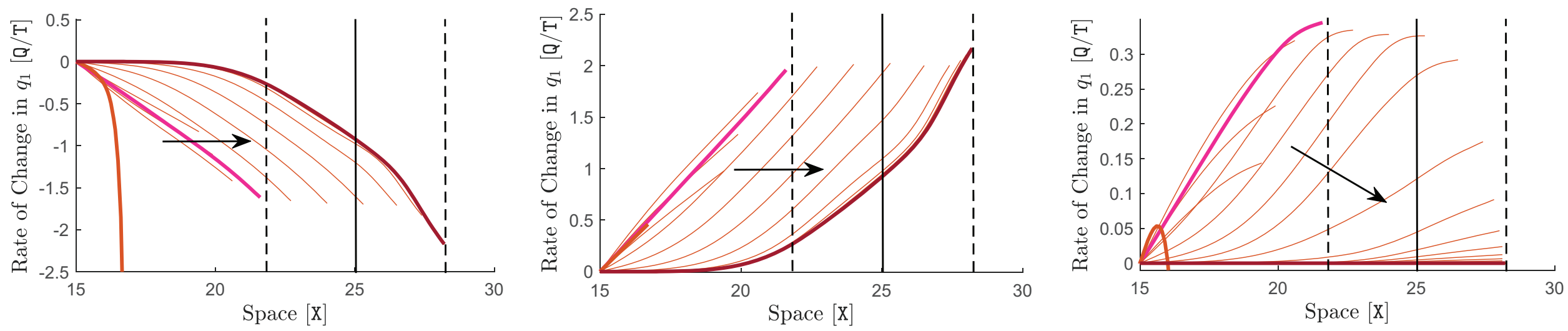

(a) Dispersal Effects **(b)** Local Effects **(c)** Total Effects

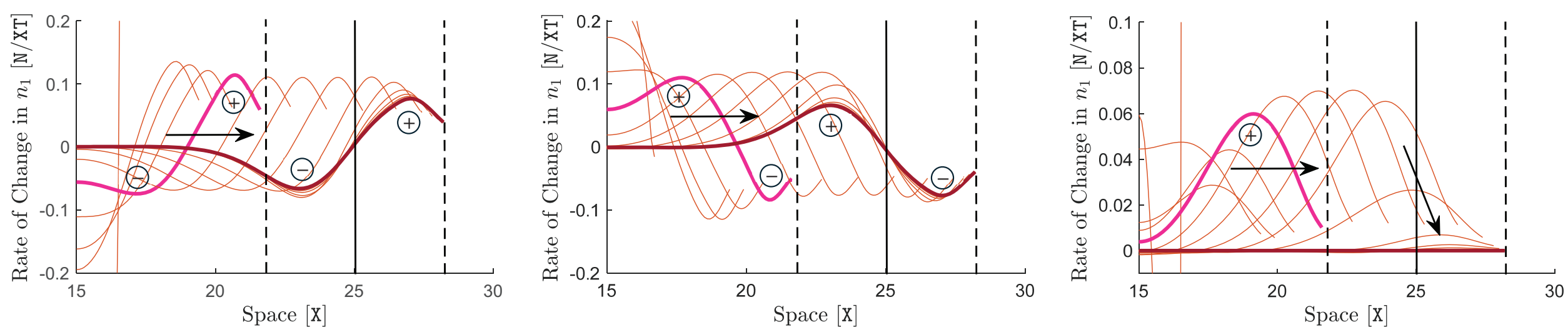

(d) Dispersal Effects **(e)** Local Effects **(f)** Total Effects

Figure S3: Effects of random-only dispersal, selection, and competition on rates of changes in trait mean and population density. The results shown here are similar to those shown in Figure 3 of the main text, but here in the absence of phenotype-optimal dispersal (only with random dispersal). Specifically, the graphs shown here complete the set of graphs shown in Figure S2 to demonstrate the effects of different components contributing to $\partial_t q_1$ (top panel) and $\partial_t n_1$ (bottom panel). As in Figure S2, the graphs are associated with the same simulation shown in the left panel of Figure 1 in the main text. Graphs (a) and (d) are the same graphs shown in Figures S2c and S2f, which are repeated here for simplicity of comparison. They show the contribution of random dispersal to $\partial_t q_1$ and $\partial_t n_1$, respectively. The local contributions of selection and competition to $\partial_t q_1$ and $\partial_t n_1$ are shown in (b) and (e), respectively. The details of the computations associated with these contributions are described in Section S2.3. Graphs (c) and (d) show the total effects, that is, they exactly show $\partial_t q_1$ and $\partial_t n_1$, respectively. The same description as in Figure 2 of the main text holds for the curve colors and the solid and dashed lines.

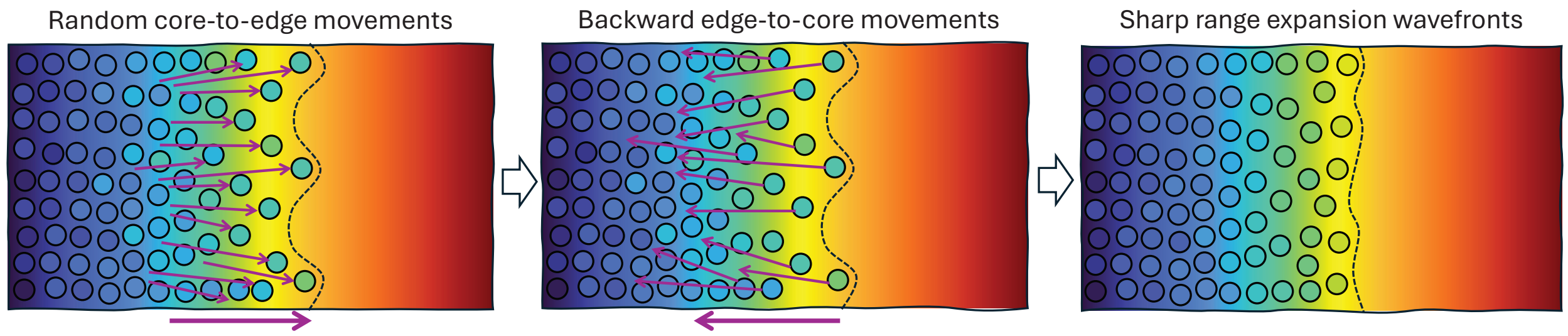

(a) Movements during range expansion

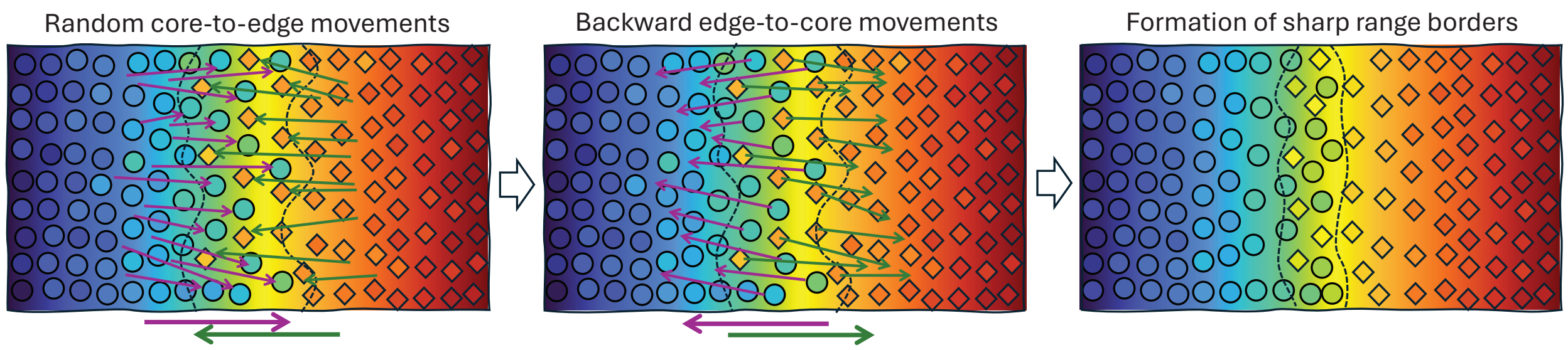

(b) Movements during border formation

Figure S4: Schematic illustration of the movements caused by random and adaptive dispersal behaviors near the species' range margins. In each graph, the predominantly core-to-edge movements caused by the random (diffusive) component of dispersal are shown on the left, and the the backward edge-to-core movements caused by matching habitat choice are shown in the middle. Although these different movement directions are shown separately here, for visualization clarity, they occur dynamically together during species' range evolution. The background color gradients indicate the gradient in optimum phenotype. Individuals of the $1st$ species are shown by circles, and individuals of the 2nd species are shown by diamonds. The individuals' color indicates their phenotype value. In (a), range expansion of a solitary species is shown. The individuals which disperse randomly from the range core to the range edge, possibly to explore new habitat, often face environments less suited for their phenotype. Following their matching habitat choice strategy, these individuals then move back to the core. These backward edge-to-core movements sharpen the range expansion wavefronts, illustrated in the diagrams on the right. Matching habitat choice results in strong adaptation (phenotype-environment match) except over a narrow region near the edge. Note that despite the sharpness of the wavefronts, the range edges shown by dashed lines keep moving forward and the species' range expands continuously. In (b), the formation of range limits between two competitively interacting species is shown. Interspecific competition and maladaptive effects of random movements tend to induce a high degree of character displacement within the region of sympatry (between the dashed lines). This can be seen in the left diagram, through the distinct difference between the color of the individuals of the two species located between the dashed lines. However, matching habitat choice pushes the individuals whose phenotype differs significantly from the optimum to move back towards their population's core. These backward movements (along with interspecific competition) eventually result in formation of sharp range limits (a narrow region of sympatry) and substantially reduced character displacement (relatively close colors).

References

D. D. Ackerly and W. K. Cornwell. A trait-based approach to community assembly: partitioning of species trait values into within- and among-community components. *Ecology Letters*, 10(2): 135–145, 2007. doi: 10.1111/j.1461-0248.2006.01006.x.

N. Barton. Adaptation at the edge of a species range. In J. Silvertown and J. Antonovics, editors, *Integrating ecology and evolution in a spatial context*, chapter 17, page 365–392. Blackwell, Oxford, 2001.

J. R. Bridle, M. Kawata, and R. K. Butlin. Local adaptation stops where ecological gradients steepen or are interrupted. *Evolutionary Applications*, 12(7):1449–1462, 2019. doi: 10.1111/eva.12789.

T. J. Case and M. L. Taper. Interspecific competition, environmental gradients, gene flow, and the coevolution of species' borders. *The American Naturalist*, 155(5):583–605, 2000. doi: 10.1086/303351.

J. B. S. Haldane. The relation between density regulation and natural selection. *Proceedings of the Royal Society of London. Series B - Biological Sciences*, 145(920):306–308, 1956. doi: 10.1098/rspb.1956.0039.

W. Hundsdorfer. Accuracy and stability of splitting with stabilizing corrections. *Applied Numerical Mathematics*, 42(1):213–233, 2002. doi: 10.1016/S0168-9274(01)00152-0.

M. Kirkpatrick and N. H. Barton. Evolution of a species' range. *The American Naturalist*, 150(1): 1–23, 1997. doi: 10.1086/286054.

E. Mayr. *Animal Species and Evolution.* Harvard University Press, Cambridge, MA, 1963.

C. M. Pease, R. Lande, and J. Bull. A model of population growth, dispersal and evolution in a changing environment. *Ecology*, 70(6):1657–1664, 1989. doi: 10.2307/1938100.

J. Polechová. Is the sky the limit? On the expansion threshold of a species' range. *PLOS Biology*, 16(6):e2005372, 2018. doi: 10.1371/journal.pbio.2005372.

J. Polechová and N. H. Barton. Limits to adaptation along environmental gradients. *Proceedings of the National Academy of Sciences*, 112(20):6401–6406, 2015. doi: 10.1073/pnas.1421515112.

F. Shirani and B. G. Freeman. Environmental "knees" and "wiggles" as strong stabilizers of species range limits set by interspecific competition. *bioRxiv*, pages 1–47, 2025. doi: 10.1101/2024.07.24.605034.

F. Shirani and J. R. Miller. Competition, trait variance dynamics, and the evolution of a species' range. *Bulletin of Mathematical Biology*, 84(3):37, 2022. doi: 10.1007/s11538-022-00990-z.

F. Shirani and J. R. Miller. Matching habitat choice and the evolution of a species' range. *Bulletin of Mathematical Biology*, 87(6):70, 2025. doi: 10.1007/s11538-025-01445-x.

C. Violle and L. Jiang. Towards a trait-based quantification of species niche. *Journal of Plant Ecology*, 2(2):87–93, 2009. doi: 10.1093/jpe/rtp007.
